# Comprehensive Analysis of Genomic Alterations in Hepatoid Adenocarcinoma of the Stomach and Identification of Clinically Actionable Alterations

**DOI:** 10.3390/cancers14163849

**Published:** 2022-08-09

**Authors:** Rongjie Zhao, Hongshen Li, Weiting Ge, Xiuming Zhu, Liang Zhu, Xiangbo Wan, Guanglan Wang, Hongming Pan, Jie Lu, Weidong Han

**Affiliations:** 1 Department of Medical Oncology, Sir Run Run Shaw Hospital, College of Medicine, Zhejiang University, Hangzhou 310016, China; 2 Cancer Institute, The Second Affiliated Hospital, College of Medicine, Zhejiang University, Hangzhou 310005, China; 3 Department of Medical Oncology, Zhejiang Provincial People’s Hospital, Hangzhou Medical College, Hangzhou 314408, China; 4 Department of Pathology, Cancer Hospital of the University of Chinese Academy of Sciences, Hangzhou 310005, China; 5 Department of Radical Oncology, The Sixth Affiliated Hospital of Sun Yat-sen University, Guangzhou 518052, China; 6 Department of Pathology, Sir Run Run Shaw Hospital, College of Medicine, Zhejiang University, Hangzhou 310016, China; 7 Department of Gastroenterology, Gongli Hospital of Shanghai Pudong New Area, Shanghai University, Shanghai 200135, China; 8 Department of Gastroenterology, The Tenth People’s Hospital of Tongji University, Shanghai 311202, China

**Keywords:** hepatoid adenocarcinoma of the stomach, whole-exome sequencing, mutagenesis mechanisms, aggressive behaviors, clinically actionable alterations

## Abstract

**Simple Summary:**

Hepatoid adenocarcinoma of the stomach (HAS) is a subset of gastric cancer (GC) histologically characterized by hepatocellular carcinoma-like foci with or without alpha-fetoprotein secretion, which is easily misdiagnosed. Genomic alterations and potential targets for this population are still largely unknown. Additionally, treatment regimens of HAS are mainly based on GC guidelines, which is not reasonable for diseases with great heterogeneity. The present study comprehensively depicts the genomic features of HAS, and they are significantly different from GC, AFP-producing GC (AFPGC), and liver hepatocellular carcinoma (LIHC). Multiple aggressive behavior-related amplificated or deleted regions in HAS are firstly reported. Moreover, reliable and practicable clinically actionable alterations for HAS are identified, providing evidence for making personalized therapy based on the genomic characteristics of HAS instead of GC.

**Abstract:**

Hepatoid adenocarcinoma of the stomach (HAS) is a rare malignancy with aggressive biological behavior. This study aimed to compare the genetic landscape of HAS with liver hepatocellular carcinoma (LIHC), gastric cancer (GC), and AFP-producing GC (AFPGC) and identify clinically actionable alterations. Thirty-eight cases of HAS were collected for whole-exome sequencing. Significantly mutated genes were identified. *TP53* was the most frequently mutated gene (66%). Hypoxia, *TNF-α*/*NFκB*, mitotic spindle assembly, DNA repair, and *p53* signaling pathways mutated frequently. Mutagenesis mechanisms in HAS were associated with spontaneous or enzymatic deamination of 5-methylcytosine to thymine and defective homologous recombination-related DNA damage repair. However, LIHC was characteristic of exposure to aflatoxin and aristolochic acid. The copy number variants (CNVs) in HAS was significantly different compared to LIHC, GC, and AFPGC. Aggressive behavior-related CNVs were identified, including local vascular invasion, advanced stages, and adverse prognosis. In 55.26% of HAS patients there existed at least one clinically actionable alteration, including *ERBB2*, *FGFR1*, *CDK4*, *EGFR*, *MET*, and *MDM2* amplifications and *BRCA1/2* mutations. *MDM2* amplification with functional *TP53* was detected in 5% of HAS patients, which was proved sensitive to *MDM2* inhibitors. A total of 10.53% of HAS patients harbored TMB > 10 muts/Mb. These findings improve our understanding of the genomic features of HAS and provide potential therapeutic targets.

## 1. Introduction

Hepatoid adenocarcinoma of the stomach (HAS) is a subset of gastric cancer (GC) histologically characterized by hepatocellular carcinoma-like foci with or without alpha-fetoprotein (AFP) secretion [1]. It is a rare cancer and has not been included in the four recently defined GC subtypes by The Cancer Genome Atlas Research Network based on molecular evaluation: EBV(+) tumors, chromosomal instability tumors, microsatellite unstable tumors, and genomically stable tumors [2]. However, previous studies reported that HAS was more aggressive in biological behavior than ordinary GC, including a higher rate of vascular invasion, regional and distant metastasis, and a strikingly worse prognosis [3,4]. Additionally, hepatocellular carcinoma-like foci in the stomach were easily misdiagnosed as metastatic liver hepatocellular carcinoma (LIHC), and wrong diagnosis and treatment decisions imposed a huge physical and economic burden on patients.

The mechanisms involved in the progression and clinically actionable targets of HAS are still largely unclear. The stomach and liver share a common embryonic origin from the foregut, making genetic progression and divergence critical factors in the development of HAS [5,6,7]. Based on immunohistochemical results, Shinichi Tsuruta MD. et al. [8] concluded that compared to GC, HAS had a relatively higher ratio of *ERBB2* expression (score 3+/2+: 21%/19%), as well as overexpression of *p16* and *IMP3*. Additionally, there were also studies showing that *TP53* was highly mutated and its abnormal function might affect HAS differentiation [1,9]. The copy number gains (CNGs) at 20q11.21–13.12 were linked to adverse biological behavior, but it was not statistically significant, which might be associated with limited information provided by panel-based sequencing technology. Therefore, it was worthwhile to further comprehensively depict the genomic characteristics of HAS by larger panel and explore the association with clinicopathological features and prognosis.

Moreover, presently, the treatment of HAS is based on GC guidelines as there is no specific and systemic treatment regimen for HAS patients. However, the morphology, biological behavior, and prognosis between HAS and GC are quite different, making it necessary to identify specific therapeutic targets. In addition, the predictive markers of immunotherapy efficacy in HAS, such as tumor mutation burden (TMB) and microsatellite instability (MSI), should be studied. The present study aimed to comprehensively compare the genomic alterations in HAS with those in LIHC, GC, and AFP-producing GC (AFPGC), and identify clinically actionable alterations.

## 2. Materials and Methods

### 2.1. Patients and Samples

This study focused on 38 cases of HAS and matched normal mucosa samples, of which 15 were collected between 2012 and 2017 from Sir Run Run Shaw Hospital, The First Affiliated Hospital of Zhejiang University, The Second Affiliated Hospital of Zhejiang University, Zhejiang Cancer Hospital, Zhejiang Provincial People’s Hospital, Shanghai Tenth People’s Hospital, and The First Affiliated Hospital of Sun Yat-sen University. The study was approved by the Ethics Committee of the affiliated Sir Run Run Shaw Hospital, School of Medicine, Zhejiang University. The other 23 cases of HAS with effective WES data were obtained from the dataset ERP128950 [10], and 31 cases of nonhepatoid differentiated patients were included as the AFPGC cohort. Additionally, we collected genomic alteration data of LIHC (364 cases) and GC (437 cases) from the TCGA database.

### 2.2. DNA Extraction and Library Construction

The genomic DNA was extracted from frozen tissues using the DNeasy Blood & Tissue Kits (Qiagen, Valencia, CA, USA). Degradation and contamination were evaluated on 1% agarose gel, and the concentration was measured using Qubit^®^ DNA Assay Kit in a Qubit^®^ 2.0 Fluorometer (Life Technologies, Carlsbad, CA, USA).

A paired-end 150 bp DNA library was constructed based on the manufacturer’s instructions (Agilent, Cedar Creek, TX, USA). Whole-exome capture was performed using Agilent SureSelect Human All Exon V6 kit (Agilent Technologies), and the captured libraries were sequenced on the Illumina Hiseq X ten platform. We then performed data quality control using fastp v0.20.1 with default parameters, including the removal of adapter contamination and low-quality reads. All the following bioinformatics were conducted on high-quality clean data. The sequencing coverage and quality statistics of each sample were summarized (Table S1).

### 2.3. Single-Nucleotide Variants (SNVs) and Copy Number Variants (CNVs) Calling

We then aligned reads to the reference genome (hg38) using Burrows–Wheeler Aligner (BWA v0.7.17). Following the best practice workflow of GATK (v4.2), we performed base recalibration with the known sites of dbSNP and 1000 Genomes, orientation bias filter, cross-sample contamination estimation, germline population filter with gnomAD, and a panel of normals to reduce systematic artifacts of sequencing and data processing. Eventually, SNVs and INDELs with the flag of “PASS” were extracted for downstream analysis, and variants were annotated by VEP (v104.3). Enrichment analysis of hallmark pathways was conducted with all mutations recorded in maf file by the OncogenicPathways function with default parameters in the maftools package. Additionally, frequency matrices generated by single 5′ and 3′ bases flanking the mutated site and NMF decomposition were conducted for de novo signature analysis by the trinucleotideMatrix and extractSignatures functions in the maftools package. Significantly mutated genes (SMG) were identified by MutSigCV (v1.41).

Copy number variant calling was executed in CNVkit (v0.9.9) with default parameters. CNV was classified into five levels, including deep deletion (CN = 0), shallow deletion (CN = 1), diploid (CN = 2), low-level amplification (CN = 3), and high-level amplification (CN ≥ 4). Loss of heterozygosity (LOH) was inferred with allelic frequency of germline heterozygous SNP by CNVkit. Significantly amplified and deleted genome regions were identified in GISTIC (v2.0). The definition of amplificated or deleted regions was as follows: deep deletion (CN = −2), shallow deletion (CN= −1), diploid (CN = 0), low-level amplification (CN = 1), and high-level amplification (CN = 2). The overall survival (OS) analysis of CNV was performed in amplification (high-level and low-level) vs. non-amplification, and deletion (deep and shallow) vs. nondeletion.

Based on the potentially drug-related targets from the oncology knowledge base (OncoKB; https://www.oncokb.org (accessed on 13 February 2022)), we systematically evaluated the landscape of clinically actionable alterations in HAS based on the results of SNVs, INDELS, high-level amplifications, and deep deletions. Microsatellite instability (MSI) status was evaluated by MANTIS (v1.0.5).

### 2.4. Statistical Analyses

Categorical variables were compared using the Chi-Squared test or Fisher’s exact test, while continuous variables were compared using the Student’s *t*-test. Overall survival analysis was calculated using the Kaplan–Meier method with log-rank test. Statistical analyses were performed using R software v4.1.1 or Graphpad Prism v7.00. *p* < 0.05 was considered statistically significant.

## 3. Results

### 3.1. Clinicopathological Characteristics of HAS

A total of 38 patients were enrolled in this study. Nearly half of the tumors were located in the antrum and were categorized as stage III-IV. HAS was typical of local vascular invasion (71.05%) and liver metastasis (21.05%). Serum AFP increase was observed in 84.21% of the patients (Table S2). The pathological features of HAS were re-evaluated by HE-stained sections under the light microscope. Different from normal gastric mucosa cells arranged in a glandular tube, hepatoid-differentiated cells had an irregular shape and were mitotically active (Figure S1A,B). They were arranged in various patterns, including nest, trabeculae, and glandular tubules (Figure S1B,C), accompanied by a tendency to infiltrate around, including local vascular invasion and liver metastasis (Figure S1D,E). Immunohistochemical results showed that 86.67% of cases were positive for AFP (Figure S1F, Table S2).

### 3.2. HAS Was Distinct in the Somatic Mutation Landscape

To compare the genomic heterogeneity of HAS, LIHC, GC, and AFPGC, we analyzed somatic mutation profiles and identified 7313 somatic alterations for 5154 genes in HAS, and the mutation proportion of frame-shift, nonsense, nonstop, and splice site was approximately 13.5% compared with 14.7% in LIHC, 22.1% in GC, and 13.0% in AFPGC, which could damage the protein structure and result in loss-of-function.

**Figure 1:**
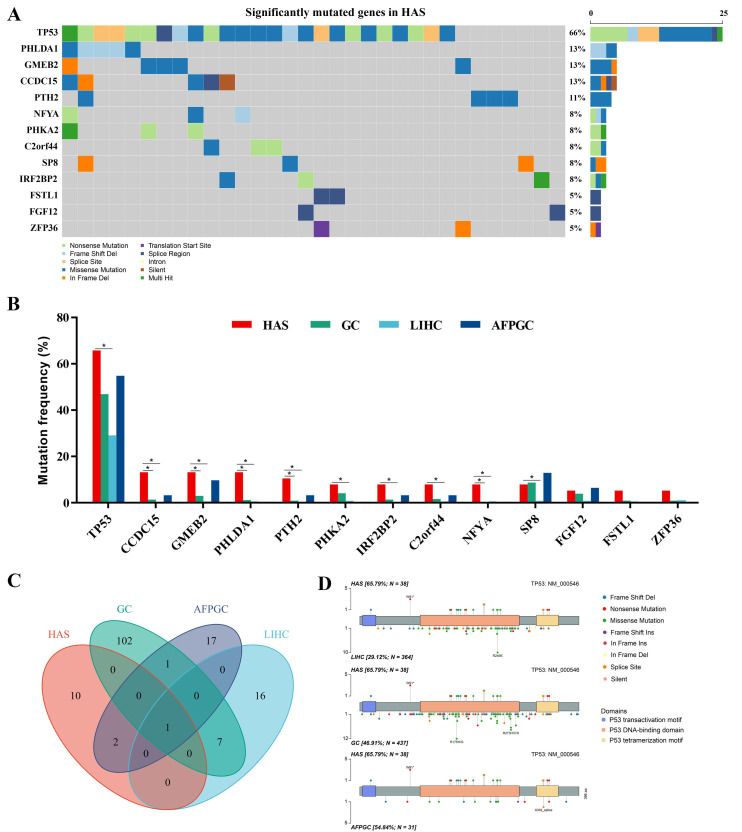
**Mutational landscape of hepatoid adenocarcinoma of the stomach (HAS).** (**A**) The significantly mutated genes (SMGs) in 38 HAS patients. Different variant types are highlighted in different colors. *p* < 0.001 is statistically significant. (**B**) Comparison of frequencies of SMGs in HAS with liver hepatocellular carcinoma (LIHC), gastric cancer (GC), and AFP-producing GC (AFPGC). * p < 0.0167, statistically significant. (**C**) Intersection of all SMGs in these cancers. (**D**) Somatic mutation site distributions of *TP53*.

The SMGs with *p* < 0.001 were shown in the oncoplot (Figure 1A, Table S3). *TP53* was the most recurrent gene (66%) in HAS, mutating more frequently than in LIHC (29%, *p* = 1.33 × 10^−5^), but similar to GC (47%, *p* = 0.028) and AFPGC (55%, *p* = 0.46) (Figure 1B and Figure S2A–C). Additionally, compared with LIHC, mutation frequencies of *CCDC15*, *CMEB2*, *PHLDA1*, *PTH2*, *PHKA2*, *IRF2BP2*, *C2orf44*, *NFYA*, and *SP8* were significantly higher in HAS (*p* < 0.0167). Nine of the thirteen SMGs (*CCDC15*, *GMEB2*, *PHLDA1*, *PTH2*, and *NFYA*) had different mutation frequencies in HAS and GC (*p* < 0.0167). Intriguingly, only one of them (*PHLDA1*) had a differentially mutated tendency in HAS and AFPGC (*p* = 0.060), indicating that HAS had more genomic differences with LIHC and GC, and shared mutational characteristics with AFPGC.

*TP53* was the only common SMG in the four cancers (Figure 1C). Further analysis showed that most of the mutation sites were located in the *p53* DNA-binding domain (Figure 1D), similar to LIHC, GC, and AFPGC. Notably, the distribution of hotspot mutations was different. Unlike missense mutation in codon 249 for LIHC, missense mutation in codon 175 and 273 for GC, and splice site mutation in codon 332 for AFPGC, the most common type for HAS was a nonsense mutation in codon 91, which could result in loss-of-function of *TP53* by reducing the expression of the protein. Protein structure changes varied widely due to different mutation types and sites, affecting the disease outcomes.

### 3.3. Mutated Pathway Analyses in HAS

**Figure 2:**
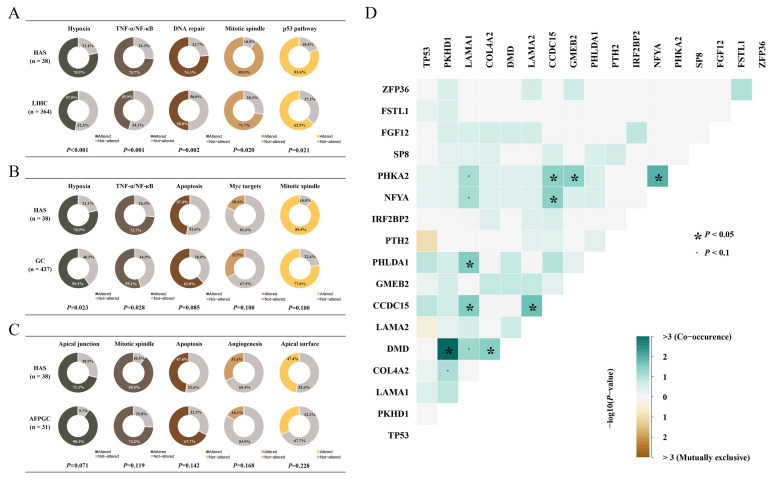
**The proportion of mutated pathways.** The proportion of patients with mutated genes in the described pathways and its differences in (**A**) HAS vs. LIHC, (**B**) HAS vs. GC, and (**C**) HAS vs. AFPGC. The curated gene sets for each pathway are obtained from the GSEA database. (**D**) Co-occurrence or mutual exclusivity patterns for the SMGs and representative genes. *p* < 0.05 is statistically significant.

Pathway enrichment analysis enhanced our understanding of the biological effects of mutations. Based on 50 hallmark gene sets from GSEA (Gene Set Enrichment Analysis), we found that multiple oncogenic pathways were frequently mutated in HAS, and part of them were distinct from those in LIHC and GC (Figure 2A,B) [11]. Mitotic spindle assembly was the most recurrently affected pathway in HAS, where 89.5% of cases (*n* = 34) had at least one mutation in this pathway, including *MYH9* (*n* = 4, 10.5%), *CDC27* (*n* = 4, 10.5%), and *TRIO* (*n* = 4, 10.5%). Other recurrently and differentially mutated pathways in HAS vs. GC and HAS vs. LIHC were as follows: hypoxia pathway with 51 mutant genes in 30 HAS patients (78.9% vs. 47.5% of LIHC, *p* < 0.001; 78.9% vs. 59.5% of GC, *p* < 0.05), *TNF-α/NF-kB* pathway with 48 mutant genes in 28 HAS patients (73.7% vs. 45.9% of LIHC, *p* = 0.001; 73.7% vs. 55.1% of GC, *p* = 0.028), DNA repair pathway with 38 mutant genes in 29 HAS patients (76.3% vs. 50.0% of LIHC, *p* = 0.002; 76.3% vs. 70.0% of GC, *p* > 0.05), and *p53* pathway with 42 mutant genes in 31 HAS patients (81.6% vs. 62.9% of LIHC, *p* = 0.021; 81.6% vs. 79.4% of GC, *p* > 0.05). However, pathway mutation frequencies between HAS and AFPGC only showed a different tendency (*p* > 0.05; Figure 2C).

*TP53* mutation was observed in 66% of HAS patients, and it was the common node of *p53*, Wnt/β-Catenin, and the DNA-repair pathway indicating that these pathways might function simultaneously in the development and progression of HAS (Figure S3). Moreover, pairwise associations between recurrent alterations were used to determine significant mutual exclusivity and co-occurrence, which identified the co-mutations of *LAMA1* in the epithelial–mesenchymal transition (EMT) pathway and *PHLDA1* in the *TNF-α*/*NF-κB* pathway, and the co-mutations of *PKHD1* in the apical surface pathway and *DMD* in the G2M checkpoint pathway, indicating that many aberrant pathways tend to co-exist. We also found that multiple genes in a pathway simultaneously mutated in a patient, such as *LAMA1*, *LAMA2*, and *COL4A2* in the EMT pathway (Figure 2D and Figure S3).

### 3.4. Mutagenesis Mechanism Heterogeneities among HAS, LIHC, GC, and AFPGC

**Figure 3:**
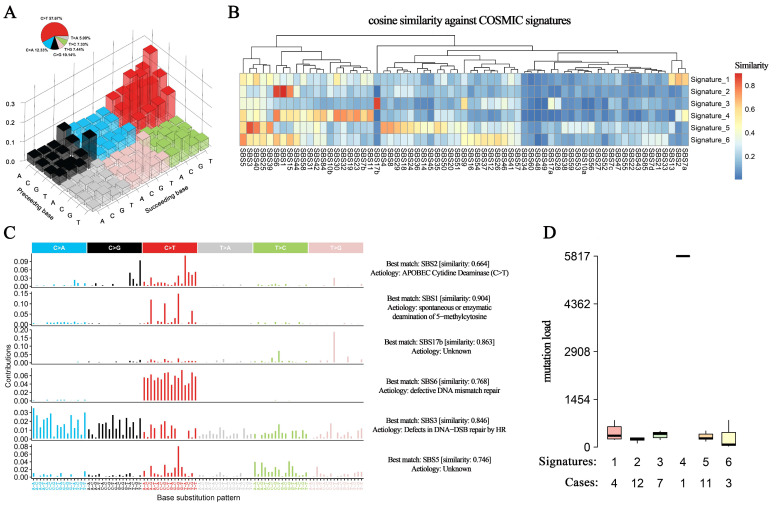
**Base substitution patterns and mutation signatures.** (**A**) A total of 96 types of base substitution patterns and their somatic mutation proportion are derived from HAS. Red: C > T, blue: C > A, black: C > G, pink: T > G, green: T > C, and grey: T > A. The height of the columns represents the proportion of each base substitution. (**B**) Six signatures inferred from these patterns are mapped to known signatures in the COSMIC database to calculate cosine similarities. (**C**) Annotations to signatures in HAS indicate potential mutagenesis mechanisms. (**D**) The number of patients and mutation load in each signature. Colors represent different signatures. N represents the number of patients attributed to each signature.

Different mutational mechanisms could induce specific combinations of mutation types. To characterize such kinds of mutational signatures, we calculated the distribution of six single-base substitutions, including C > A, C > G, C > T, T > A, T > C, and T > G, and found that the dominant substitution pattern among patients with HAS was C > T changes (57.67% of all SNVs), followed by C > A (12.33%), C > G (10.14%), T > G (7.44%), T > C (7.33%), and T > A (5.09%) (Figure 3A), which was quite similar to that in GC and AFPGC. In contrast, in LIHC, the proportions of these base substitutions were relatively evenly distributed (Figure S4A–C).

Further analysis of the context of bases immediately 3′ and 5′ to the mutated base generated a total of 96 different mutation patterns with the above six types of substitutions, and we identified six mutation signatures in HAS patients and calculated their correlations with known mutation signatures from the Catalogue of Somatic Mutations in Cancer (COSMIC) database (v3; Figure 3B) [12]. The first signature was correlated with the SBS2 COSMIC signature (cosine similarity = 0.664), *APOBEC*-induced mutagenesis, which was typical of enrichment of C > T or C > G changes at TCW motifs, in which W was A or T (Figure 3C) and was involved in the local hypermutation phenomenon. The second signature showed a high cosine similarity with the SBS1, which was associated with the endogenous mutation process of spontaneous or enzymatic deamination of 5-methylcytosine to thymine, and served as a cell division/mitotic clock that controls division rates (cosine similarity = 0.904). The fourth signature was correlated with the SBS6 (defective DNA mismatch repair, cosine similarity = 0.768), and the patients with this mutation suffered a strikingly higher mutation load than the other patients (Figure 3D), which might be associated with increased genomic instability. Similarly, the fifth signature had a high correlation with defective homologous recombination-related DNA damage repair (cosine similarity = 0.846; Figure 3C). The aetiology in the third and sixth signatures (SBS17b and SBS5) was still unclear and remained to be further annotated by COSMIC.

All these SBS COSMIC signatures in HAS except SBS2 were shared in AFPGC, and most of them were associated with defective DNA repair indicating the instable genomic features in AFPGC (Figure S4C). In GC, the mutagenesis mechanisms in half of the patients (48.3%) were characterized by polymerase epsilon exonuclease domain mutations and spontaneous or enzymatic deamination of 5-methylcytosine to thymine (Figure S4A). In contrast, exposure to aflatoxin and aristolochic acid (cosine similarity > 0.9) was considered one of the main factors in LIHC (Figure S4B), which was consistent with the current reports on the causes of liver cancer [13,14].

### 3.5. Copy Number Variants Correlated with Outcomes of HAS

**Figure 4:**
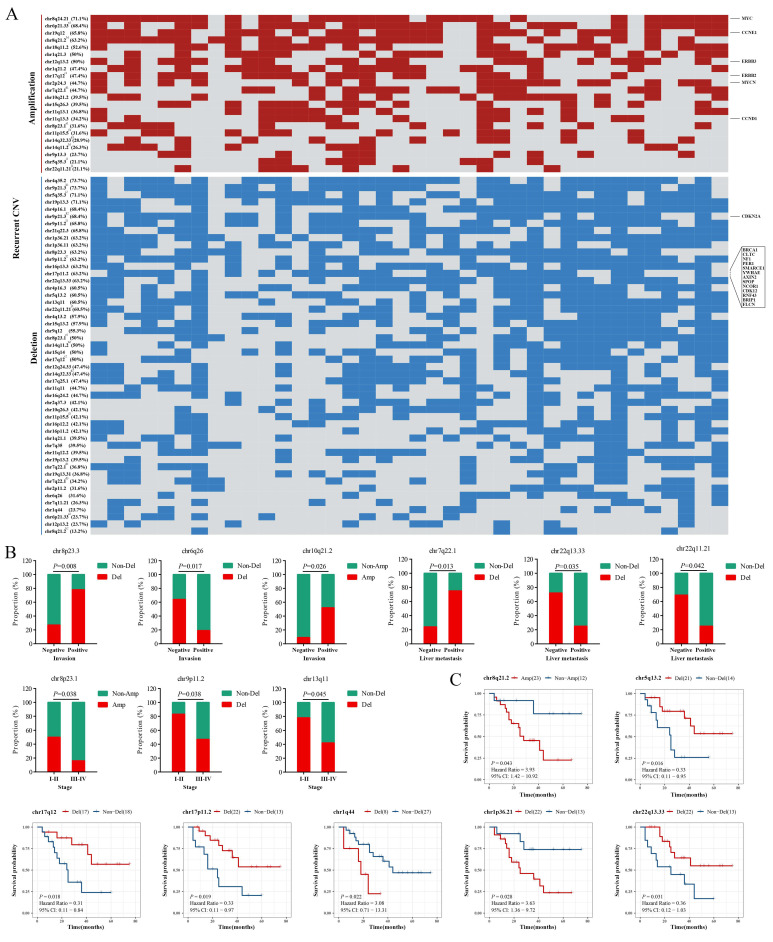
**Copy number variants (CNVs) in HAS.** (**A**) The heat map shows the recurrent CNVs in HAS. Oncogenes and tumor suppressor genes (TSG) in these regions are marked at the right of the heat map, and the list is provided by the COSMIC database. Red: amplification, blue: deletion. The repeatedly occurred regions marked with a-l are located in different wide peak limits which were recorded in Table S4. The percentage in each row represents the number of samples with the CNVs. Mutated regions are significantly related to (**B**) local vascular invasion, liver metastasis, and advanced stages and (**C**) prognosis. *p* < 0.05 is statistically significant.

We then evaluated the copy number variants in HAS and found extensive CNVs, including 22 significant amplifications and 50 deletions (*q*-value < 0.05; Figure 4A). Regions such as chr4q35.2, chr5q35.3, chr8q24.21, chr9p21.3, and chr19p13.3 were frequently altered in over 70% of patients. We exclusively focused on the aggressive biological behavior of HAS and analyzed its correlation with CNVs. Deletions in chr7q22.1 and chr8p23.3, and amplification in chr10q21.2 were positively associated with local vascular invasion or liver metastasis, while deletions in chr6q26, chr9p11.2, chr13q11, chr22q11.21, chr22q13.33, and amplifications in chr8p23.1 were negatively associated with local vascular invasion, liver metastasis, or advanced stages (*p* < 0.05; Figure 4B). Additionally, patients with amplifications in chr8q21.2, or deletions in chr1q44 and chr1p36.21, had shorter overall survival, while deletions in chr5q13.2, chr17q12, chr17p11.2, and chr22q13.33 had better overall survival (*p* < 0.05; Figure 4C). Additionally, some variants showed potential correlations with these adverse outcomes (*p* > 0.05; Figures S5 and S6). Similar to HAS, LIHC, GC, and AFPGC also harbored several significantly altered regions (Figure S7A,B), and six of them (amplifications in chr8q24.21, chr11q13.3, and chr19q12, and deletions in chr1p36.11, chr14q32.33, and chr19p13.3) were commonly shared by the four types of cancers (Figure S7C,D), suggesting that these variants might be an early molecular event [15,16].

CNVs in oncogenes and tumor suppressor genes (TSGs) could cause cell growth disorder and contribute to the aberrant proliferation of tumor cells. In the COSMIC gene list, we identified a total of six oncogenes and 14 TSGs located in these frequently altered regions (Figure 4A). OS analysis of all oncogenes and TSGs recorded in the COSMIC database showed that deletions of four TSGs (*SBDS*, *FANCC*, *PTCH1*, and *XPA*; Figure S8A–D) and amplifications of six oncogenes (*PIK3CB*, *GATA2*, *WWTR1*, *MUC4*, *RARA*, and *AFF3*; Figure S9A–F) predicted a shorter OS (*p* < 0.05). Additionally, deletions of the left four TSGs (PPP6C, WNK2, CASP3, and CD274; Figure S8E–H) and amplifications of the thirteen oncogenes (*BCL6*, *ETV5*, *LPP*, *SOX2*, *ERBB2*, *MECOM*, *FOXR1*, *HEY1*, *CCR7*, *IL7R*, *MYC*, *TRRAP*, and *CTNNA2*; Figure S9G–S) were potentially associated with a shorter OS (*p* > 0.05).

### 3.6. Overview of Clinically Actionable Alterations

**Figure 5:**
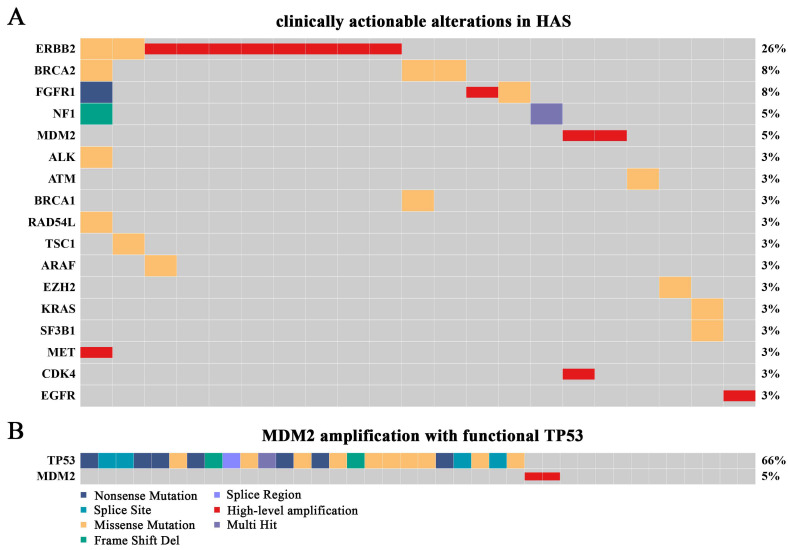
**Overview of clinically actionable alterations in HAS.** (**A**) The landscape of potentially targetable alterations based on the results of SNVs, INDELS, high-level amplifications, and deep deletions. Different variant types are highlighted in different colors. (**B**) Patients with high-level amplification of *MDM2* and functional *TP53*.

Clinically actionable alterations analysis showed that a total of 17 targets from 21 patients (55.26%) were identified (Figure 5A). Specifically, 17 patients (44.74%) had no clinically actionable alteration, 14 (36.84%) had one, and 7 (18.42%) had at least two targets. *ERBB2* amplification was the most recurrent clinically actionable alteration, followed by amplifications in *MDM2*, *FGFR1*, *MET*, *CDK4,* and *EGFR*, and oncogenic mutations in genes such as *BRCA1/2*, *NF1,* and *ALK*. Clinically, several *ERBB2*-amplified GC patients could benefit from trastuzumab target therapy. Therefore, we were greatly interested in the higher proportion of *ERBB2* amplification in HAS (21.05%), while it was approximately 14% in GC, 0.6% in LIHC, and 25.8% in AFPGC, indicating that anti-*ERBB2* treatment in HAS is also a promising option (Figure S10A–C). Additionally, *MDM2* amplification with functional *TP53* was found for the first time in HAS patients. Multiple clinical trials showed that solid tumors with *MDM2* amplification as well as wild-type or functional *TP53* were sensitive to milademetan and ALRN-6924 [17,18,19], an inhibitor of *MDM2*, suggesting that HAS patients might also benefit from the therapy since the proportion of the genotype added up to 5% (Figure 5B; Table S5).

Increasing evidence showed that TMB and MSI status were potential biomarkers for predicting patients’ response to the PD-1/PD-L1 inhibitors in various cancers [20,21]. However, the therapeutic value of TMB and MSI in HAS has not been reported before. In this study, TMB of HAS ranked fifth across 33 types of cancers from TCGA cohorts with a median TMB of 3.41 muts/Mb (range: 0.22–69.28 muts/Mb; Figure S11), indicating obvious heterogeneity among patients. Four HAS patients harbored TMB > 10 muts/Mb, which might be sensitive to immunotherapy. Notably, TMB in HAS and AFPGC was significantly higher than LIHC (range: 0.03–34.16 muts/Mb; *p* < 0.001). There were no significant differences in HAS vs. GC (range: 0.03–268.66 muts/Mb; *p* = 0.53) and HAS vs. AFPGC (range: 0–30.88 muts/Mb; *p* = 0.97). Microsatellite instability analysis showed all HAS patients were microsatellite stable (MSS; Table S6).

## 4. Discussion

HAS is a kind of tumor located in the stomach and typical of hepatoid differentiation with or without the secretion of AFP, which has a strong ability of invasion and metastasis [3,4]. Currently, genomic alterations and potential treatment targets for HAS are still largely unknown due to the rarity of these cases. In the present study, in more than half of HAS cases there existed local vascular invasion, and 21.05% of HAS cases metastasized to the liver, turning into advanced stages. The genetic landscape showed that HAS was obviously different compared to LIHC and GC in terms of recurrent SNVs, pathways, mutagenesis mechanisms, and CNVs, but shared mutational characteristics with AFPGC to some extent. Notably, multiple significantly amplified or deleted genome regions were firstly found involved in local vascular invasion, liver metastasis, advanced stages, and overall survival of HAS, which could enhance the understanding of aggressive biological behaviors. A total of 55.26% of HAS patients harbored at least one clinically actionable alteration, providing evidence for personalized treatment.

Ji et al. depicted the recurrently mutated genes and regions in HAS, but the correlations of these variants with the aggressive behaviors of HAS such as invasion, metastasis, stages, and adverse prognosis were not researched [9]. In addition, due to the overlap in morphology and location of HAS, GC, LIHC, and AFPGC, we made a comprehensive comparison of genomic characteristics, and it improved our knowledge about HAS and was helpful to further studies.

Among the SMGs, *TP53* mutation was the most frequent variant. It was the shared node in *p53*, *Wnt*/*β-Catenin*, and the DNA-repair pathway, indicating that multiple oncogenic pathways co-exist and function simultaneously in most HAS patients, which might promote tumorigenesis and invasion of HAS [22,23]. Additionally, six of the SMGs were more highly mutated in HAS than in both LIHC and GC, including *PHLDA1*, *GMEB2*, *CCDC15*, *PTH2*, *NFYA*, and *C2orf44*. Mutation frequencies of genes mentioned above were similar in HAS and AFPGC except *PHLDA1* (*p* = 0.060), high expression of which could inhibit the survival and metastasis of gastric cancer and served as a TSG [24]. *PHLDA1* downregulation was proved to promote acquisition and maintenance of drugs resistance targeting receptor tyrosine kinase, which might suppress the effect of trastuzumab treatment on *ERBB2* amplification cancers [25].

The mutation spectrum in the context of 96 types of base constitution varied a lot, and the pattern could reflect mutagenic mechanisms in tumorigenesis. The deficiency of DNA mismatch repair was common in all the cancers, but the preference for the TCW mutation pattern mediated by *APOBEC* was only identified in HAS, indicating genomic specificity [26]. For LIHC, chemical carcinogenesis was considered the main mutation initiation factor, including exposure to aflatoxin and aristolochic acid [27], which have been widely accepted and ensure the reliability of the results.

Increasing evidence reveals that CNVs induced by various endogenous and exogenous stimuli existed in tumor tissues and were critical in genetic heterogeneity [28], transformation of precancerous lesions [29], and treatment efficacy [30]. There were six mutated regions detected in all of these cancers, including amplifications in chr8q24.21, chr11q13.3, and chr19q12, and deletions in chr1p36.11, chr14q32.33, and chr19p13.3, which might play an important role in cancer development and progression. For example, chr8q24.21 amplification was frequent in advanced GC [31], and involved in liver metastasis of rectal adenocarcinoma [32]. chr19q12 amplification was prevalent in multiple tumor types [33]. Consistently, in 65.8% of HAS cases there existed the amplification of chr19q12. Amplification of the *CCNE1* locus in this region was synthetic lethal related, targeted by *PKMYT1* kinase inhibition, and it was also a promising target in AFPGC, which warranted further research in HAS [10,33]. Deleted regions-associated studies were mainly involved in gastrointestinal defects, Peutz-Jeghers syndrome (19p13.3), and immunodeficiency (14q32.33) [34,35,36,37]. There were 32 regions specifically amplificated or deleted in HAS (Figure S7C,D), and the amplification of chr22q11.21 and deletions of chr1p36.21, chr7q11.21, chr7q22.1, chr9q12 as well as chr12q24.33 predicted a tendency of shorter OS. Intriguingly, Farshidfar et al. demonstrated that chr22q11.21 amplification in melanoma was strongly associated with inferior survival, metastasis, and downregulation of immunotherapy response-related genes [38], indicating its important role in HAS. Additionally, chr10q21.2 amplification was linked with precursor B-cell acute lymphoblastic leukemia of childhood [39], and in this study we found it was associated with local vascular invasion. These results provided insights into the mechanisms underlying aggressive behaviors.

The mutation landscape of HAS was significantly distinct from GC, including SNVs, mutational patterns, pathways, and CNVs. Therefore, it was necessary to understand HAS comprehensively and develop treatment strategies based on its genomic features. The previous study considered MAT2A and AHCY as potential targets to HAS by clustering and comparing the transcription profiles of HAS and TCGA-GC. However, there must be batch effects between two groups from two different cohorts, which were not corrected and might notably affect the reliability of the result [9]. We identified targets of four levels which were more reliable and practicable, including FDA-approved drugs, standard card, clinical evidence, and biological evidence [40]. Our data showed that 55.26% of HAS patients carried at least one potentially targetable alteration. *ERBB2* amplification was present in more than 20% of HAS patients who had a shorter OS tendency (Figure S9K), which was similar to GC [41]. However, it created novel agents and opportunities. *ERBB2*-targeted therapy greatly improved the prognosis of GC patients with *ERBB2* amplification [42,43]. Such benefits were also observed beyond gastric and breast cancer, including in bladder, biliary, and colon cancers [43]. Therefore, it might also serve as a promising treatment choice for HAS patients. Additionally, *MDM2* was a negative regulator of the tumor suppressor *TP53* [44]. Inhibiting the interaction between wild-type *p53* and *MDM2* was one of the main strategies to develop compounds targeting dysfunctional *p53* for cancer treatment [45,46], which has been introduced into clinical trials, showing that solid tumor patients with *MDM2* amplification as well as wild-type or functional *TP53* could benefit from *MDM2* inhibitors, milademetan, and ALRN-6924 [17,18,19,47]. The proportion of the genotype added up to 5% in HAS patients indicating an *MDM2* inhibitor is also a promising option.

Additionally, mutations in *BRCA1* (3%) and *BRCA2* (8%) were detected in HAS. An increasing number of clinical trials have shown that various malignancies with *BRCA* mutation may potentially be sensitive to PARP inhibitors [48], which offered a potential treatment option for HAS. High TMB and high microsatellite instability are conventionally regarded as useful biomarkers to predict immunotherapy response. At present, the definition of high and low levels of TMB is still controversial, but the most widely accepted standard is 10 muts/Mb [49]. In the present study, 10.52% of HAS patients harbored high TMB, and the proportions in TCGA-GC, TCGA-LIHC, and AFPGC cohorts were 21.5%, 1.6%, and 3.2% respectively. All HAS patients were in MSS condition, suggesting that only a small number of HAS patients might benefit from immunotherapy. This requires further validation.

There was a limitation in our study. The incidence of HAS was very low, resulting in a small sample size, which may lead to some deviations in the results. Some novel variations related to the malignant characteristics of HAS were identified. Further functional validation of the variations may have improved the reliability of the conclusion, but the scarcity of samples made the experimental verification difficult.

## 5. Conclusions

HAS has its specific molecular variant characteristics, and it is different from LIHC, GC, and AFPGC. We have identified progression-associated alterations, and provided an overview of clinically actionable alterations of HAS, including *ERBB2* amplification and *MDM2* amplification with functional *TP53*, which are helpful for establishing a treatment scheme based on its own genetic background.

## Data Availability

The WES data generated in this study are available in the National Genomics Data Center under accession number HRA001552. The accessible link is as follows: https://ngdc.cncb.ac.cn/gsa-human/s/B4fS79Vl (accessed on 1 January 2022). Other data that support the findings of this study are available from the corresponding author upon request.

## Supplementary Materials

The following are available online at https://www.mdpi.com/article/10.3390/cancers14163849/s1, Figure S1: Representative pathological sections, Figure S2: Mutational landscapes of LIHC, GC, and AFPGC, Figure S3: Representative mutated pathways in HAS, Figure S4: Base substitution patterns and mutation signatures, Figure S5: Potentially aggressive behavior-related regions, Figure S6: Potentially overall survival-related regions, Figure S7: The landscape of significantly altered regions, Figure S8: The role of tumor suppressor genes (TSGs) in HAS overall survival, Figure S9: The role of oncogenes in HAS overall survival, Figure S10: The amplification of *ERBB2* in GC, LIHC, and AFPGC, Figure S11: Distribution of Tumor Mutational Burden (TMB), Table S1. The statistics of the whole-exome sequencing data, Table S2: Clinical characteristics of 38 HAS patients, Table S3: Significantly mutated genes in HAS patients, Table S4: Annotation of the repeatedly occurred regions, Table S5: SNV and CNV of *TP53* in HAS patients, Table S6: Microsatellite statuses of HAS patients.

## References

[1] Wang Y., Sun L., Li Z., Gao J., Ge S., Zhang C., Yuan J., Wang X., Li J., Lu Z. (2019). Hepatoid adenocarcinoma of the stomach: A unique subgroup with distinct clinicopathological and molecular features. Gastric Cancer.

[2] The Cancer Genome Atlas Research Network (2014). Comprehensive molecular characterization of gastric adenocarcinoma. Nature.

[3] Xie Y., Zhao Z., Li P., Wang Y., Guo C., Wang X., Tang W., Liu Q., Lu N., Xue L. (2015). Hepatoid adenocarcinoma of the stomach is a special and easily misdiagnosed or missed diagnosed subtype of gastric cancer with poor prognosis but curative for patients of pN0/1: The experience of a single center. Int. J. Clin. Exp. Med..

[4] Zeng X.Y., Yin Y.P., Xiao H., Zhang P., He J., Liu W.Z., Gao J.B., Shuai X.M., Wang G.B., Wu X.L. (2018). Clinicopathological Characteristics and Prognosis of Hepatoid Adenocarcinoma of the Stomach: Evaluation of a Pooled Case Series. Curr. Med. Sci..

[5] Shoenfeld Y. (1989). Experimental and induced animal models of systemic lupus erythematosus and Sjogren’s syndrome. Curr. Opin. Rheumatol..

[6] Fujii H., Ichikawa K., Takagaki T., Nakanishi Y., Ikegami M., Hirose S., Shimoda T. (2003). Genetic evolution of alpha fetoprotein producing gastric cancer. J. Clin. Pathol..

[7] Akiyama S., Tamura G., Endoh Y., Fukushima N., Ichihara Y., Aizawa K., Kawata S., Motoyama T. (2003). Histogenesis of hepatoid adenocarcinoma of the stomach: Molecular evidence of identical origin with coexistent tubular adenocarcinoma. Int. J. Cancer.

[8] Tsuruta S., Ohishi Y., Fujiwara M., Ihara E., Ogawa Y., Oki E., Nakamura M., Oda Y. (2019). Gastric hepatoid adenocarcinomas are a genetically heterogenous group; most tumors show chromosomal instability, but MSI tumors do exist. Hum. Pathol..

[9] Liu Z., Wang A., Pu Y., Li Z., Xue R., Zhang C., Xiang X., E J.-Y., Bu Z., Bai F. (2021). Genomic and transcriptomic profiling of hepatoid adenocarcinoma of the stomach. Oncogene.

[10] Lu J., Ding Y., Chen Y., Jiang J., Chen Y., Huang Y., Wu M., Li C., Kong M., Zhao W. (2021). Whole-exome sequencing of alpha-fetoprotein producing gastric carcinoma reveals genomic profile and therapeutic targets. Nat. Commun..

[11] Liberzon A., Birger C., Thorvaldsdottir H., Ghandi M., Mesirov J.P., Tamayo P. (2015). The Molecular Signatures Database (MSigDB) hallmark gene set collection. Cell Syst..

[12] Alexandrov L., Kim J., Haradhvala N., Huang M., Tian Ng A., Wu Y., Boot A., Covington K., Gordenin D., Bergstrom E. (2020). The repertoire of mutational signatures in human cancer. Nature.

[13] Huet-Duvillier G., Gomes V., Tetaert D., Mathon P., Boersma A., Degand P. (1988). Trypanosoma brucei brucei: Variability in the association of some variant surface glycoproteins. Exp. Parasitol..

[14] McGlynn K.A., Petrick J.L., El-Serag H.B. (2021). Epidemiology of Hepatocellular Carcinoma. Hepatology.

[15] Kang J.U. (2014). Chromosome 8q as the most frequent target for amplification in early gastric carcinoma. Oncol. Lett..

[16] Shi Z.Z., Shang L., Jiang Y.Y., Hao J.J., Zhang Y., Zhang T.T., Lin D.C., Liu S.G., Wang B.S., Gong T. (2013). Consistent and differential genetic aberrations between esophageal dysplasia and squamous cell carcinoma detected by array comparative genomic hybridization. Clin. Cancer Res..

[17] Saleh M.N., Patel M.R., Bauer T.M., Goel S., Falchook G.S., Shapiro G.I., Chung K.Y., Infante J.R., Conry R.M., Rabinowits G. (2021). Phase 1 Trial of ALRN-6924, a Dual Inhibitor of MDMX and MDM2, in Patients with Solid Tumors and Lymphomas Bearing Wild-Type *TP53*. Clin. Cancer Res..

[18] Konopleva M., Martinelli G., Daver N., Papayannidis C., Wei A., Higgins B., Ott M., Mascarenhas J., Andreeff M. (2020). MDM2 inhibition: An important step forward in cancer therapy. Leukemia.

[19] Takahashi S., Fujiwara Y., Nakano K., Shimizu T., Tomomatsu J., Koyama T., Ogura M., Tachibana M., Kakurai Y., Yamashita T. (2021). Safety and pharmacokinetics of milademetan, a MDM2 inhibitor, in Japanese patients with solid tumors: A phase I study. Cancer Sci..

[20] Chao J., Fuchs C.S., Shitara K., Tabernero J., Muro K., Van Cutsem E., Bang Y.J., De Vita F., Landers G., Yen C.J. (2021). Assessment of Pembrolizumab Therapy for the Treatment of Microsatellite Instability-High Gastric or Gastroesophageal Junction Cancer Among Patients in the KEYNOTE-059, KEYNOTE-061, and KEYNOTE-062 Clinical Trials. JAMA Oncol..

[21] Valero C., Lee M., Hoen D., Zehir A., Berger M.F., Seshan V.E., Chan T.A., Morris L.G.T. (2021). Response Rates to Anti-PD-1 Immunotherapy in Microsatellite-Stable Solid Tumors With 10 or More Mutations per Megabase. JAMA Oncol..

[22] Huang C.K., Yu T., de la Monte S.M., Wands J.R., Derdak Z., Kim M. (2015). Restoration of Wnt/β-catenin signaling attenuates alcoholic liver disease progression in a rat model. J. Hepatol..

[23] Vaughan C.A., Singh S., Subler M.A., Windle J.J., Inoue K., Fry E.A., Pillappa R., Grossman S.R., Windle B., Yeudall W.A. (2021). The oncogenicity of tumor-derived mutant p53 is enhanced by the recruitment of PLK3. Nat. Commun..

[24] Wang L., Shen J., Jiang Y. (2018). Circ_0027599/PHDLA1 suppresses gastric cancer progression by sponging miR-101-3p.1. Cell Biosci..

[25] Fearon A.E., Carter E.P., Clayton N.S., Wilkes E.H., Baker A.M., Kapitonova E., Bakhouche B.A., Tanner Y., Wang J., Gadaleta E. (2018). PHLDA1 Mediates Drug Resistance in Receptor Tyrosine Kinase-Driven Cancer. Cell Rep..

[26] Law E.K., Levin-Klein R., Jarvis M.C., Kim H., Argyris P.P., Carpenter M.A., Starrett G.J., Temiz N.A., Larson L.K., Durfee C. (2020). APOBEC3A catalyzes mutation and drives carcinogenesis in vivo. J. Exp. Med..

[27] Yang J.D., Hainaut P., Gores G.J., Amadou A., Plymoth A., Roberts L.R. (2019). A global view of hepatocellular carcinoma: Trends, risk, prevention and management. Nat. Rev. Gastroenterol. Hepatol..

[28] Kim H., Nguyen N.P., Turner K., Wu S., Gujar A.D., Luebeck J., Liu J., Deshpande V., Rajkumar U., Namburi S. (2020). Extrachromosomal DNA is associated with oncogene amplification and poor outcome across multiple cancers. Nat. Genet..

[29] Killcoyne S., Gregson E., Wedge D.C., Woodcock D.J., Eldridge M.D., de la Rue R., Miremadi A., Abbas S., Blasko A., Kosmidou C. (2020). Genomic copy number predicts esophageal cancer years before transformation. Nat. Med..

[30] Jogo T., Nakamura Y., Shitara K., Bando H., Yasui H., Esaki T., Terazawa T., Satoh T., Shinozaki E., Nishina T. (2021). Circulating Tumor DNA Analysis Detects FGFR2 Amplification and Concurrent Genomic Alterations Associated with FGFR Inhibitor Efficacy in Advanced Gastric Cancer. Clin. Cancer Res..

[31] Arakawa N., Sugai T., Habano W., Eizuka M., Sugimoto R., Akasaka R., Toya Y., Yamamoto E., Koeda K., Sasaki A. (2017). Genome-wide analysis of DNA copy number alterations in early and advanced gastric cancers. Mol. Carcinog..

[32] Zhou H.T., Shi Z.Z., Zhou Z.X., Jiang Y.Y., Hao J.J., Zhang T.T., Shi F., Xu X., Wang M.R., Zhang Y. (2013). Genomic changes in rectal adenocarcinoma associated with liver metastasis. Cancer Biomark..

[33] Gallo D., Young J.T.F., Fourtounis J., Martino G., Alvarez-Quilon A., Bernier C., Duffy N.M., Papp R., Roulston A., Stocco R. (2022). CCNE1 amplification is synthetic lethal with PKMYT1 kinase inhibition. Nature.

[34] Geier C.B., Piller A., Eibl M.M., Ciznar P., Ilencikova D., Wolf H.M. (2017). Terminal 14q32.33 deletion as a novel cause of agammaglobulinemia. Clin. Immunol..

[35] Gruber S.B., Entius M.M., Petersen G.M., Laken S.J., Longo P.A., Boyer R., Levin A.M., Mujumdar U.J., Trent J.M., Kinzler K.W. (1998). Pathogenesis of adenocarcinoma in Peutz-Jeghers syndrome. Cancer Res..

[36] Khalili A., Yadegari A.H., Delavari S., Yazdani R., Abolhassani H. (2021). Disseminated Intravascular Coagulation Associated with Large Deletion of Immunoglobulin Heavy Chain. Iran. J. Allergy Asthma Immunol..

[37] Serra G., Felice S., Antona V., Di Pace M.R., Giuffre M., Piro E., Corsello G. (2022). Cardio-facio-cutaneous syndrome and gastrointestinal defects: Report on a newborn with 19p13.3 deletion including the MAP 2 K2 gene. Ital. J. Pediatr..

[38] Farshidfar F., Rhrissorrakrai K., Levovitz C., Peng C., Knight J., Bacchiocchi A., Su J., Yin M., Sznol M., Ariyan S. (2022). Integrative molecular and clinical profiling of acral melanoma links focal amplification of 22q11.21 to metastasis. Nat. Commun..

[39] Prasad R.B., Hosking F.J., Vijayakrishnan J., Papaemmanuil E., Koehler R., Greaves M., Sheridan E., Gast A., Kinsey S.E., Lightfoot T. (2010). Verification of the susceptibility loci on 7p12.2, 10q21.2, and 14q11.2 in precursor B-cell acute lymphoblastic leukemia of childhood. Blood.

[40] Chakravarty D., Gao J., Phillips S.M., Kundra R., Zhang H., Wang J., Rudolph J.E., Yaeger R., Soumerai T., Nissan M.H. (2017). OncoKB: A Precision Oncology Knowledge Base. JCO Precis. Oncol..

[41] Bayrak M., Olmez O.F., Kurt E., Cubukcu E., Evrensel T., Kanat O., Manavoglu O. (2013). Prognostic significance of c-erbB2 overexpression in patients with metastatic gastric cancer. Clin. Transl. Oncol..

[42] Bang Y.J., Van Cutsem E., Feyereislova A., Chung H.C., Shen L., Sawaki A., Lordick F., Ohtsu A., Omuro Y., Satoh T. (2010). Trastuzumab in combination with chemotherapy versus chemotherapy alone for treatment of HER2-positive advanced gastric or gastro-oesophageal junction cancer (ToGA): A phase 3, open-label, randomised controlled trial. Lancet.

[43] Meric-Bernstam F., Johnson A.M., Dumbrava E.E.I., Raghav K., Balaji K., Bhatt M., Murthy R.K., Rodon J., Piha-Paul S.A. (2019). Advances in HER2-Targeted Therapy: Novel Agents and Opportunities Beyond Breast and Gastric Cancer. Clin. Cancer Res..

[44] Hou H., Sun D., Zhang X. (2019). The role of MDM2 amplification and overexpression in therapeutic resistance of malignant tumors. Cancer Cell Int..

[45] Duffy M.J., Synnott N.C., O′Grady S., Crown J. (2022). Targeting p53 for the treatment of cancer. Semin. Cancer Biol..

[46] Levine A.J. (2020). p53: 800 million years of evolution and 40 years of discovery. Nat. Rev. Cancer.

[47] Stein E.M., DeAngelo D.J., Chromik J., Chatterjee M., Bauer S., Lin C.C., Suarez C., de Vos F., Steeghs N., Cassier P.A. (2022). Results from a First-in-Human Phase I Study of Siremadlin (HDM201) in Patients with Advanced Wild-Type TP53 Solid Tumors and Acute Leukemia. Clin. Cancer Res..

[48] Poveda A., Floquet A., Ledermann J.A., Asher R., Penson R.T., Oza A.M., Korach J., Huzarski T., Pignata S., Friedlander M. (2021). Olaparib tablets as maintenance therapy in patients with platinum-sensitive relapsed ovarian cancer and a BRCA1/2 mutation (SOLO2/ENGOT-Ov21): A final analysis of a double-blind, randomised, placebo-controlled, phase 3 trial. Lancet Oncol..

[49] Chida K., Kawazoe A., Kawazu M., Suzuki T., Nakamura Y., Nakatsura T., Kuwata T., Ueno T., Kuboki Y., Kotani D. (2021). A Low Tumor Mutational Burden and PTEN Mutations Are Predictors of a Negative Response to PD-1 Blockade in MSI-H/dMMR Gastrointestinal Tumors. Clin. Cancer Res..

