# Supplementary material for: Comprehensive Analysis of Genomic Alterations in Hepatoid Adenocarcinoma of the Stomach and Identification of Clinically Actionable Alterations
Source: Cancers (Basel). 2022 Aug 9;14(16):3849. doi: 10.3390/cancers14163849 (PMC9405706; doi:10.3390/cancers14163849)
Supplement: Supplementary file 1 [file cancers-14-03849-s001.zip › cancers-1787982-Supplementary.pdf]

*Supplementary Materials*

# Comprehensive Analysis of Genomic Alterations in Hepatoid Adenocarcinoma of the Stomach and Identification of Clinically Actionable Alterations

**Rongjie Zhao, Hongshen Li, Weiting Ge, Xiuming Zhu, Liang Zhu, Xiangbo Wan, Guanglan Wang, Hongming Pan, Jie Lu and Weidong Han**

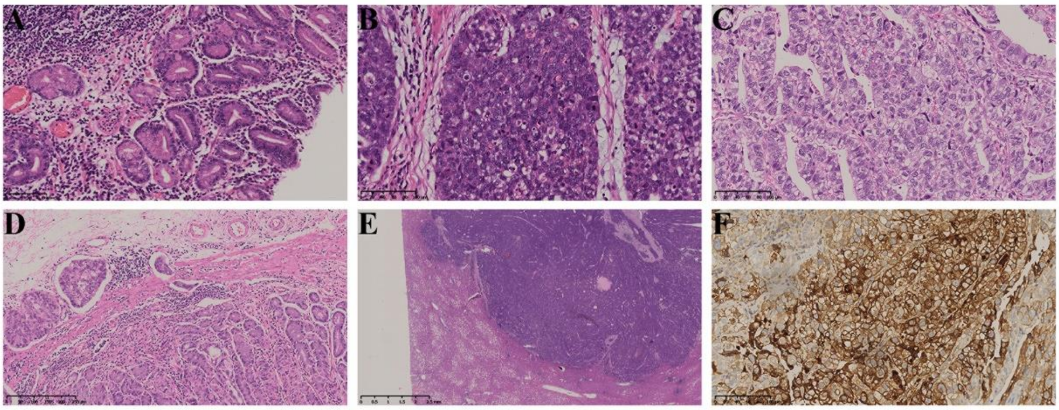

**Figure S1. Representative pathological sections. (A)** Normal gastric mucosa. **(B)** Hepatoid-differentiated cells are irregular in shape and arranged in nest, **(C)** trabeculae, and glandular tubules. **(D)** Cancer thrombus. **(E)** Liver metastasis. **(F)** Positive expression of AFP.

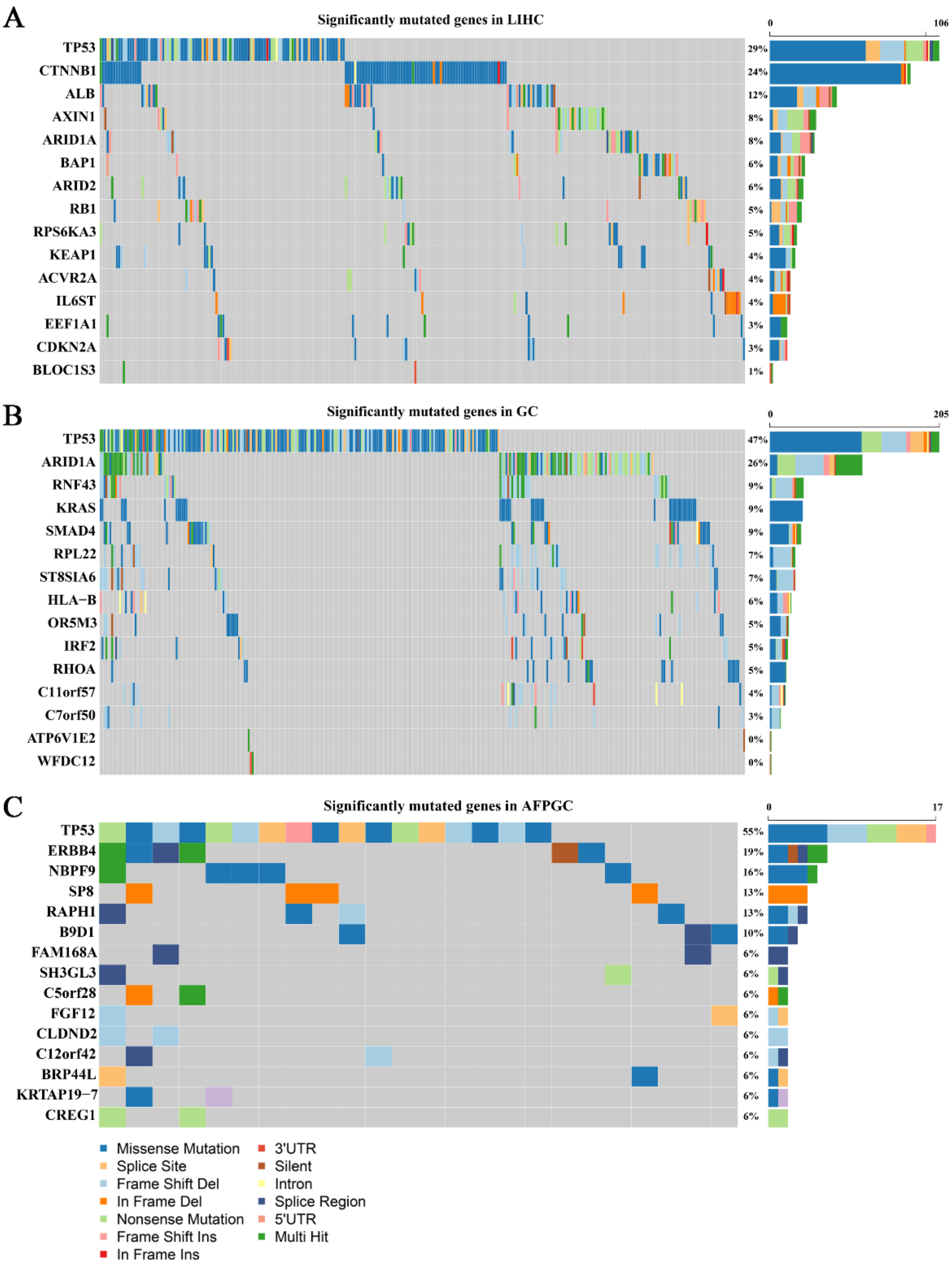


**Figure S2. Mutational landscapes of LIHC, GC and AFPGC.** The top 15 significantly mutated genes (SMGs) in **(A)** LIHC, **(B)** GC and **(C)** AFPGC. Different variant types are highlighted in different colors. $P$<0.001 is statistically significant.

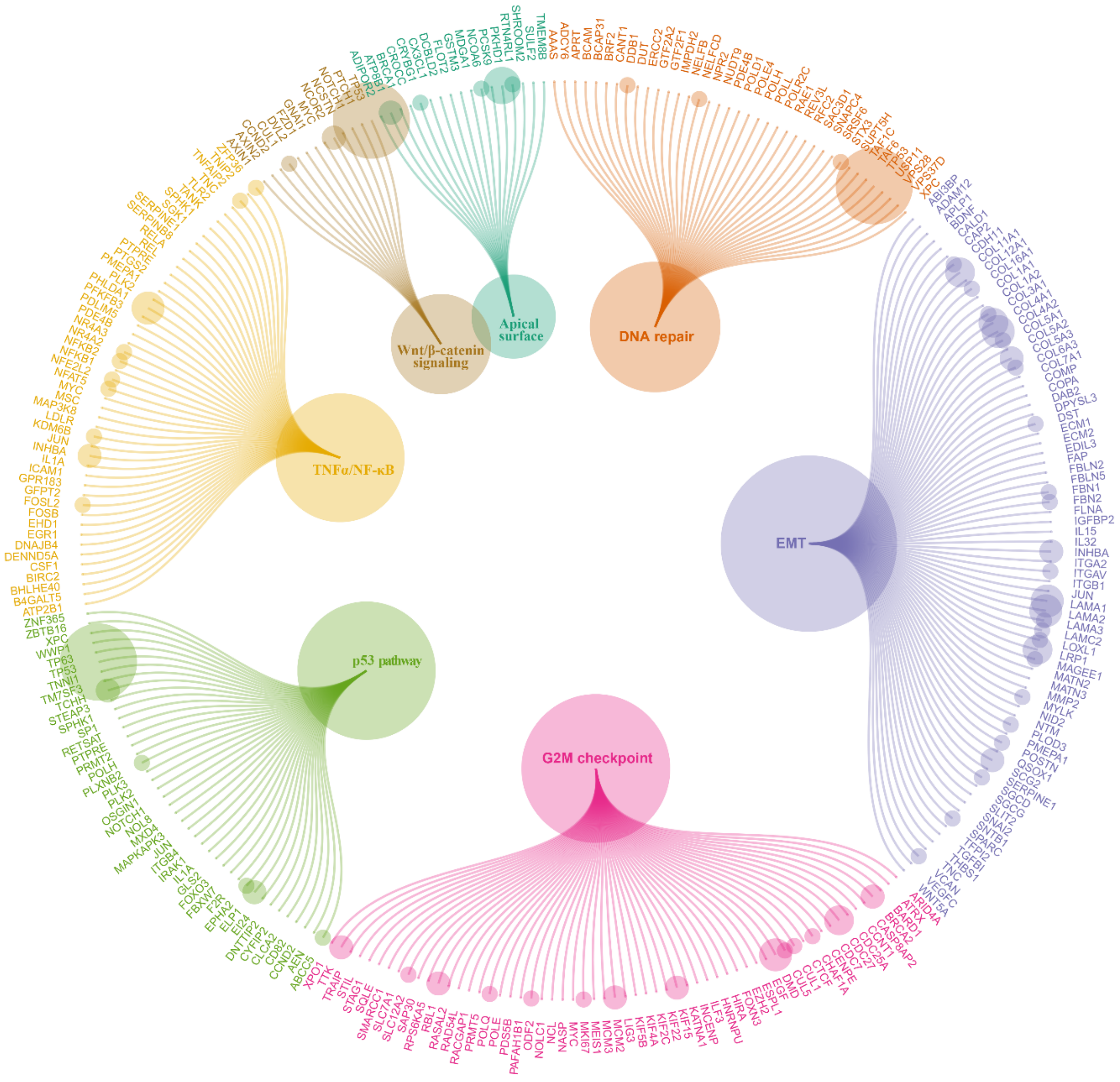


**Figure S3. Representative mutated pathways in HAS.** The size of node near each gene represents the mutation frequency.

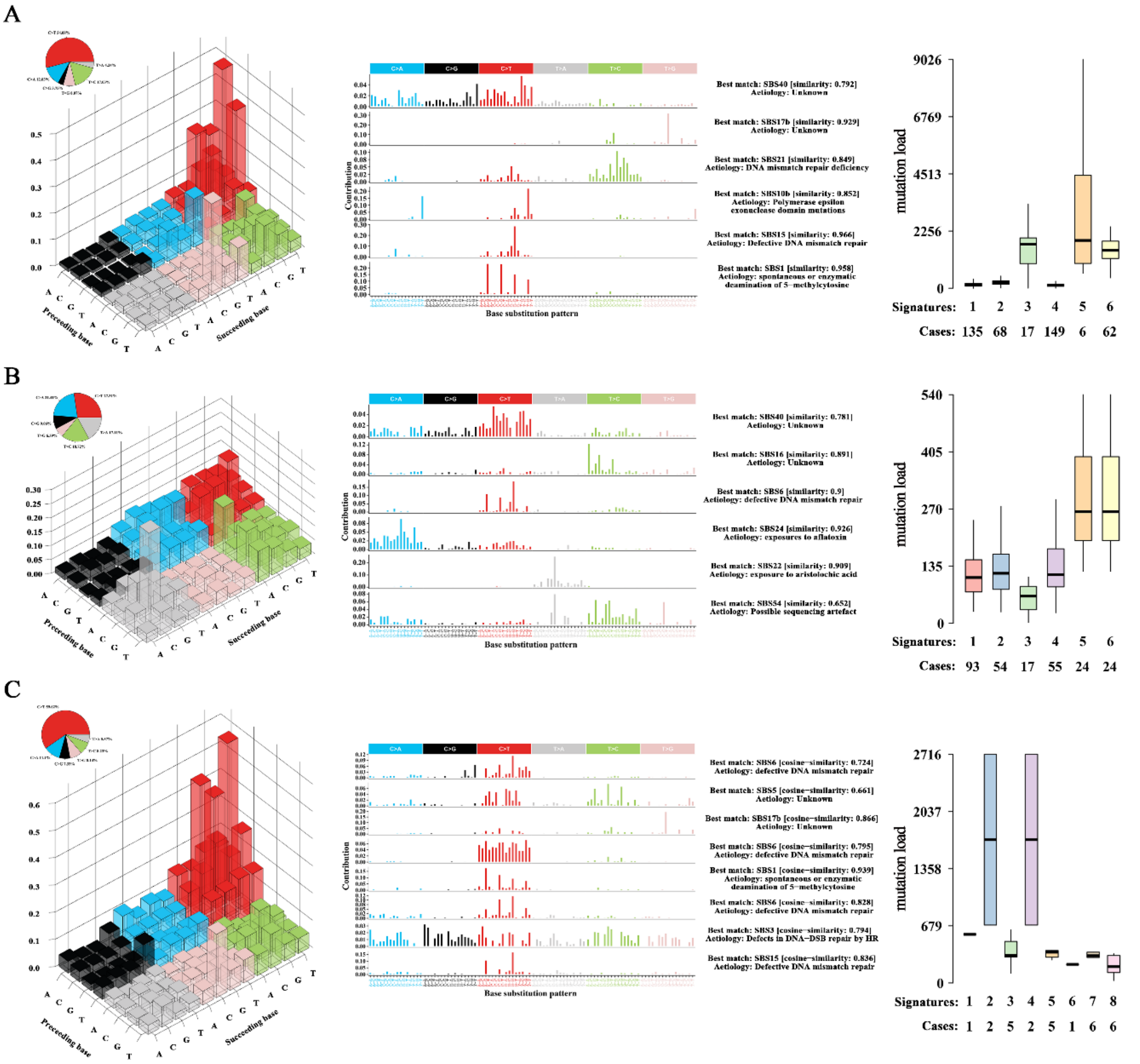


**Figure S4. Base substitution patterns and mutation signatures.** 96 types of base substitution patterns, signatures inferred from these patterns, and the distribution of patients and mutation load in each signature in **(A)** GC, **(B)** LIHC, and **(C)** AFPGC.

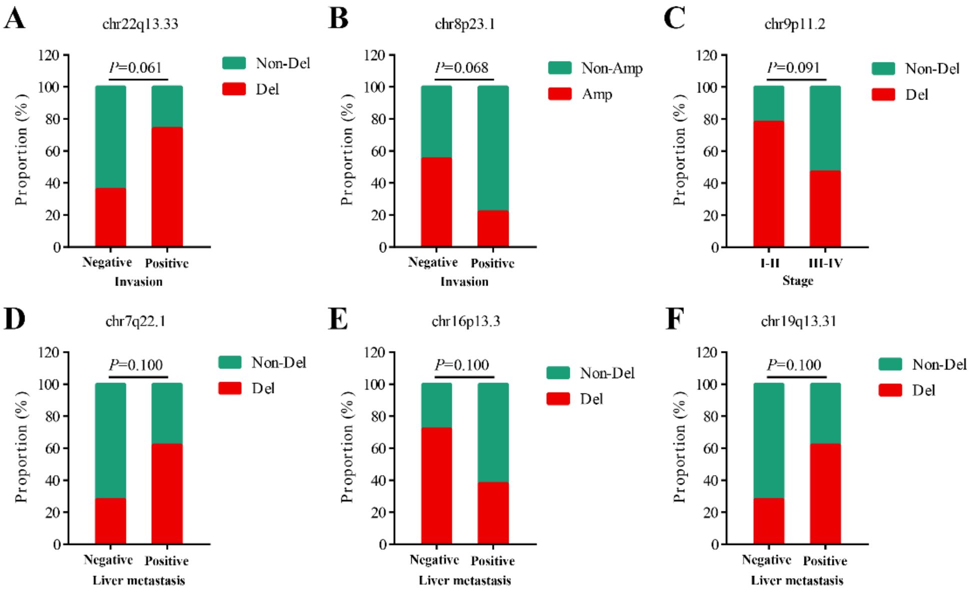


**Figure S5.** Potentially aggressive behavior-related regions.

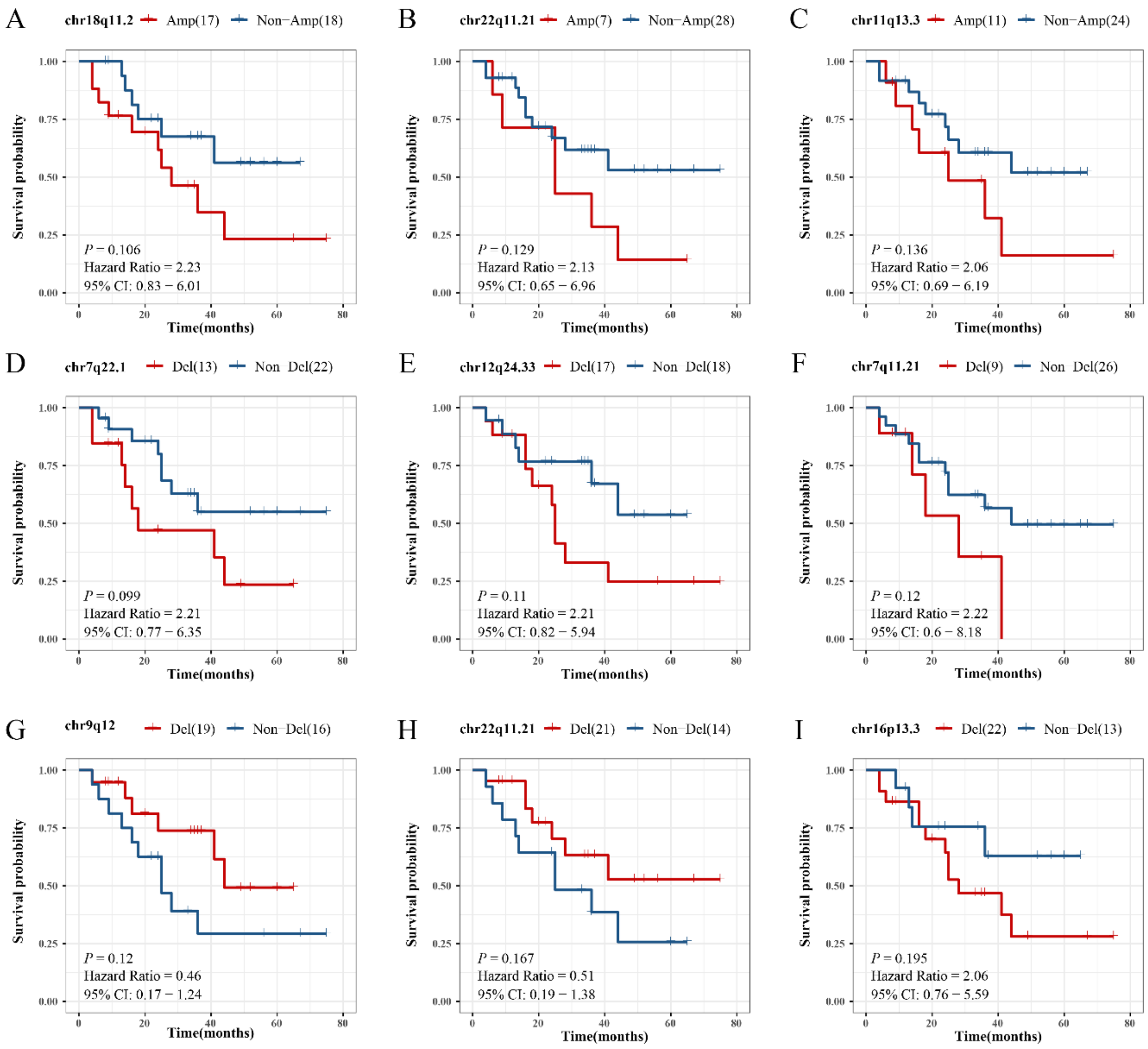


**Figure S6.** Potentially overall survival-related regions.

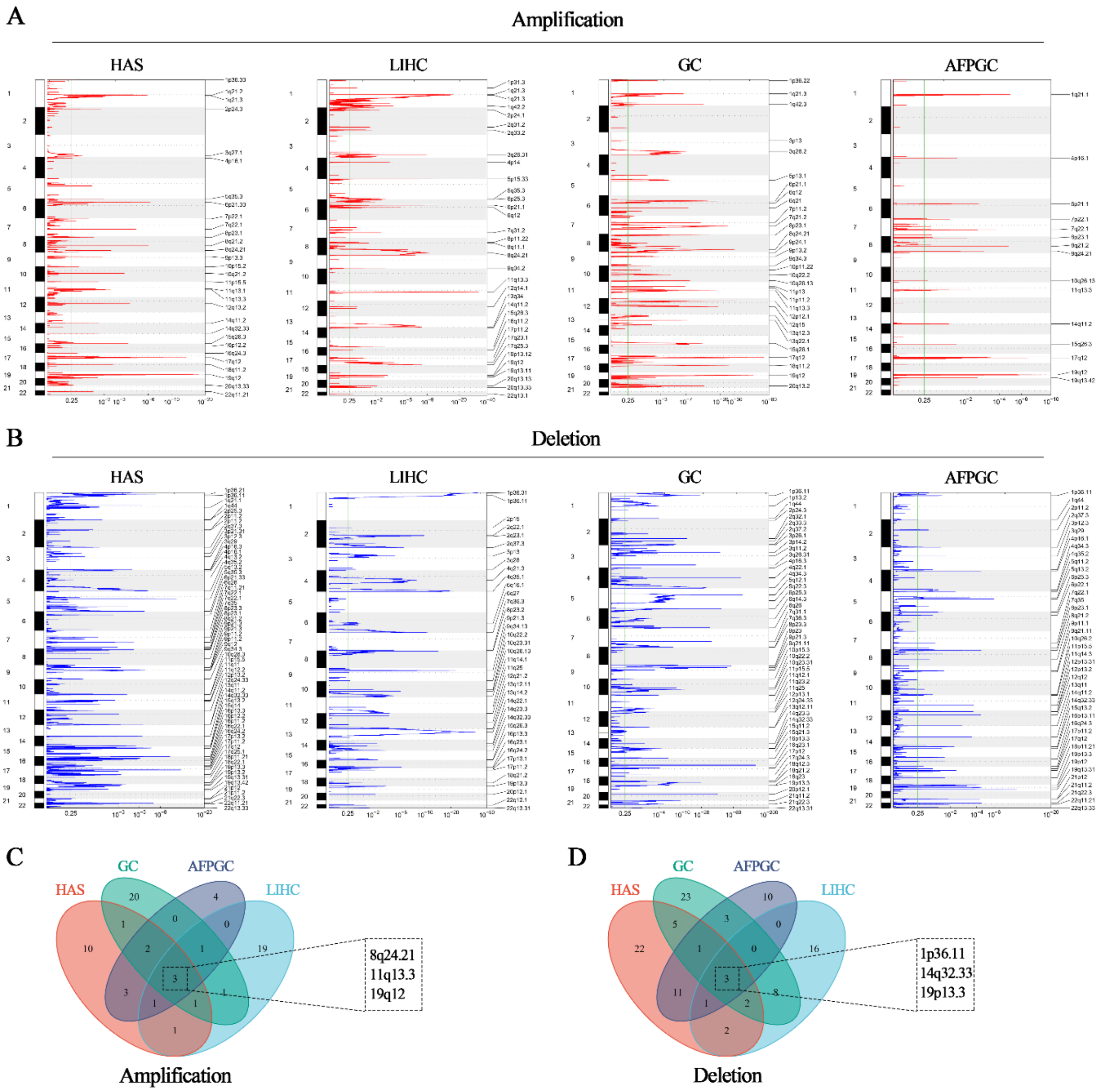


**Figure S7. The landscape of significantly altered regions.** The distribution of **(A)** amplified or **(B)** deleted regions in HAS, LIHC, GC and AFPGC. Red: amplification; Blue: deletion. The overlap in **(C)** amplified regions and **(D)** deleted regions.

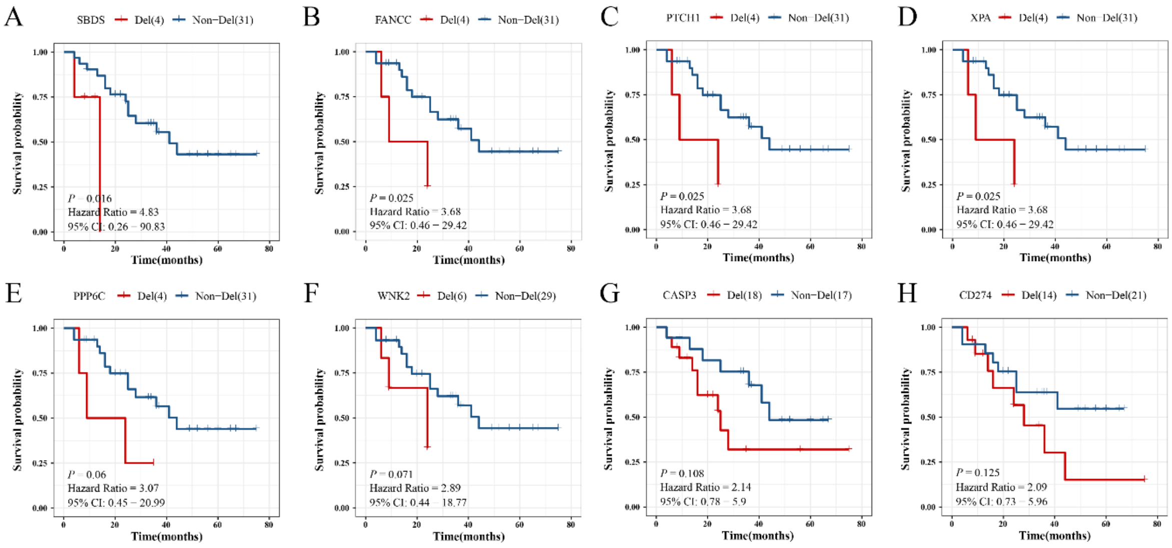

**Figure S8.** The role of tumor suppressor genes (TSGs) in HAS overall survival.

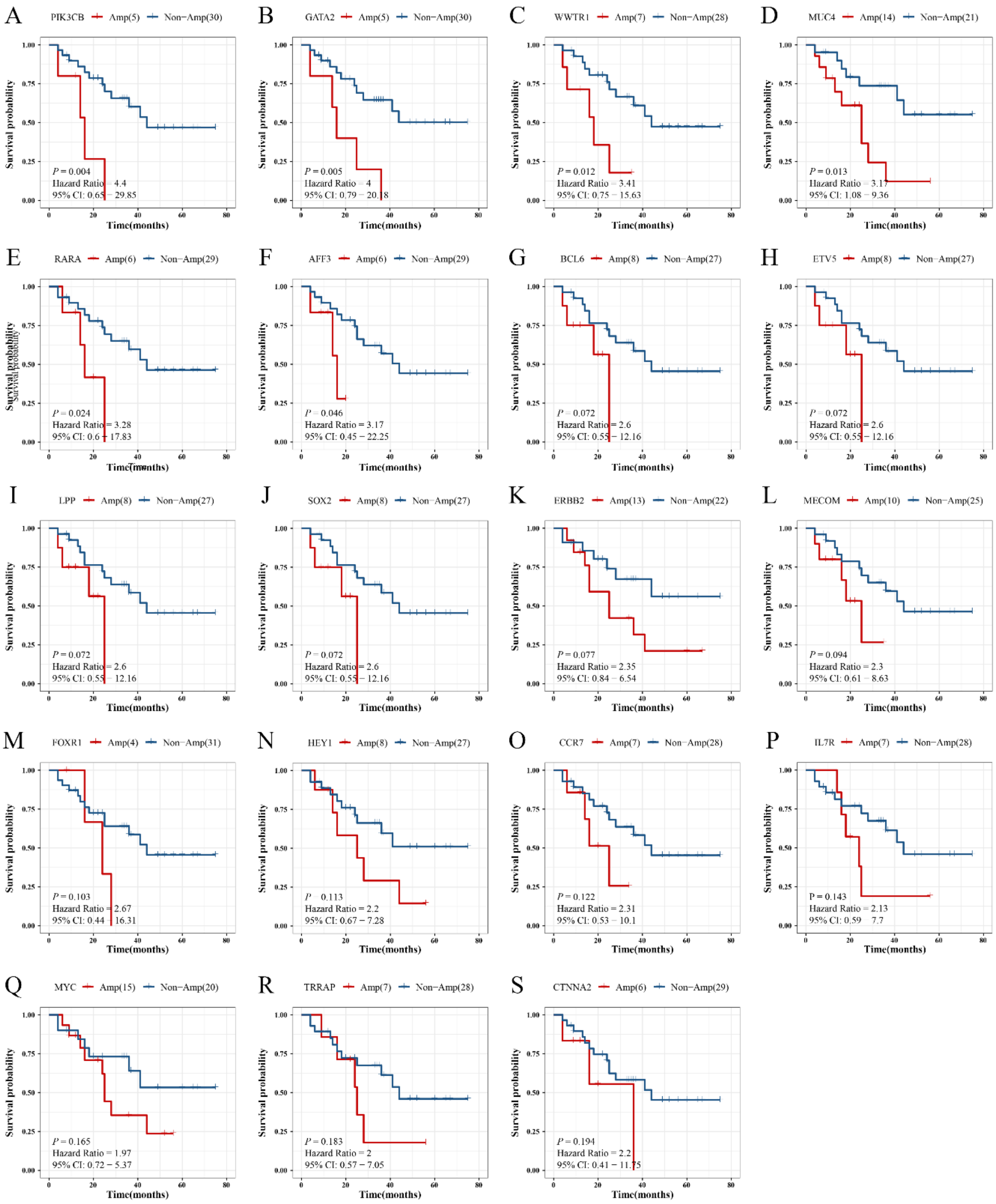


**Figure S9.** The role of oncogenes in HAS overall survival.

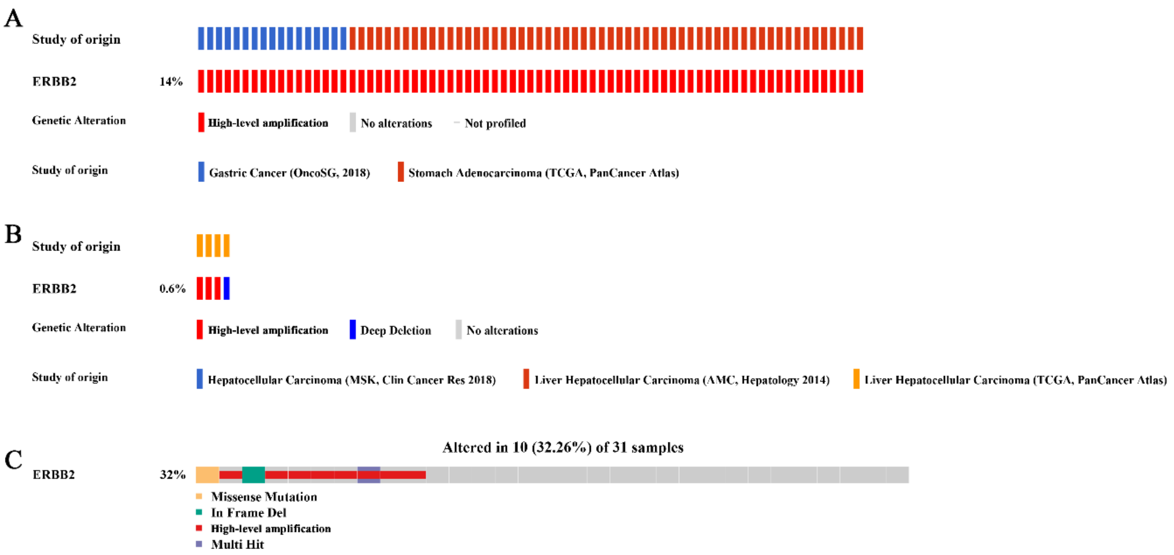


**Figure S10. The amplification of ERBB2 in GC, LIHC and AFPGC.** The proportion of high-level *ERBB2* amplification in **(A)** GC and **(B)** LIHC from cBioPortal. Unaltered samples (grey) are not shown. Red: amplification; Blue: deletion. The proportion of high-level *ERBB2* amplification in **(C)** AFPGC.

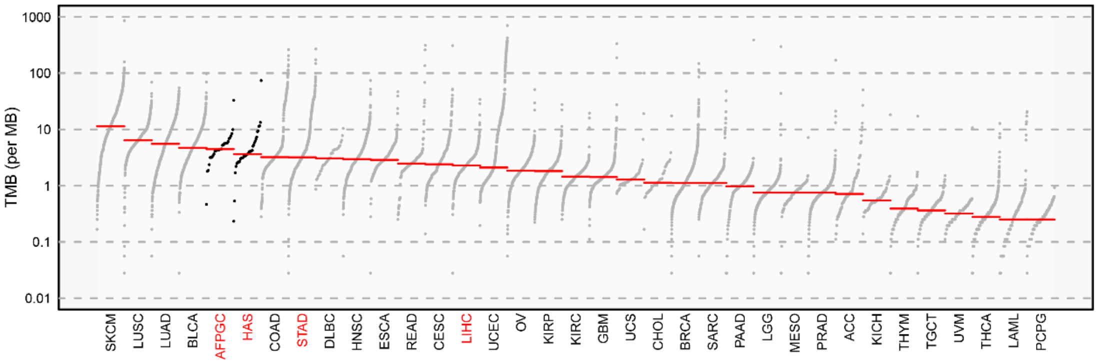


**Figure S11.** Distribution of Tumor Mutational Burden (TMB).
