# Supplementary figures and images for: Comprehensive Analysis of Genomic Alterations in Hepatoid Adenocarcinoma of the Stomach and Identification of Clinically Actionable Alterations
Source: Cancers (Basel). 2022 Aug 9;14(16):3849. doi: 10.3390/cancers14163849 (PMC9405706; doi:10.3390/cancers14163849)

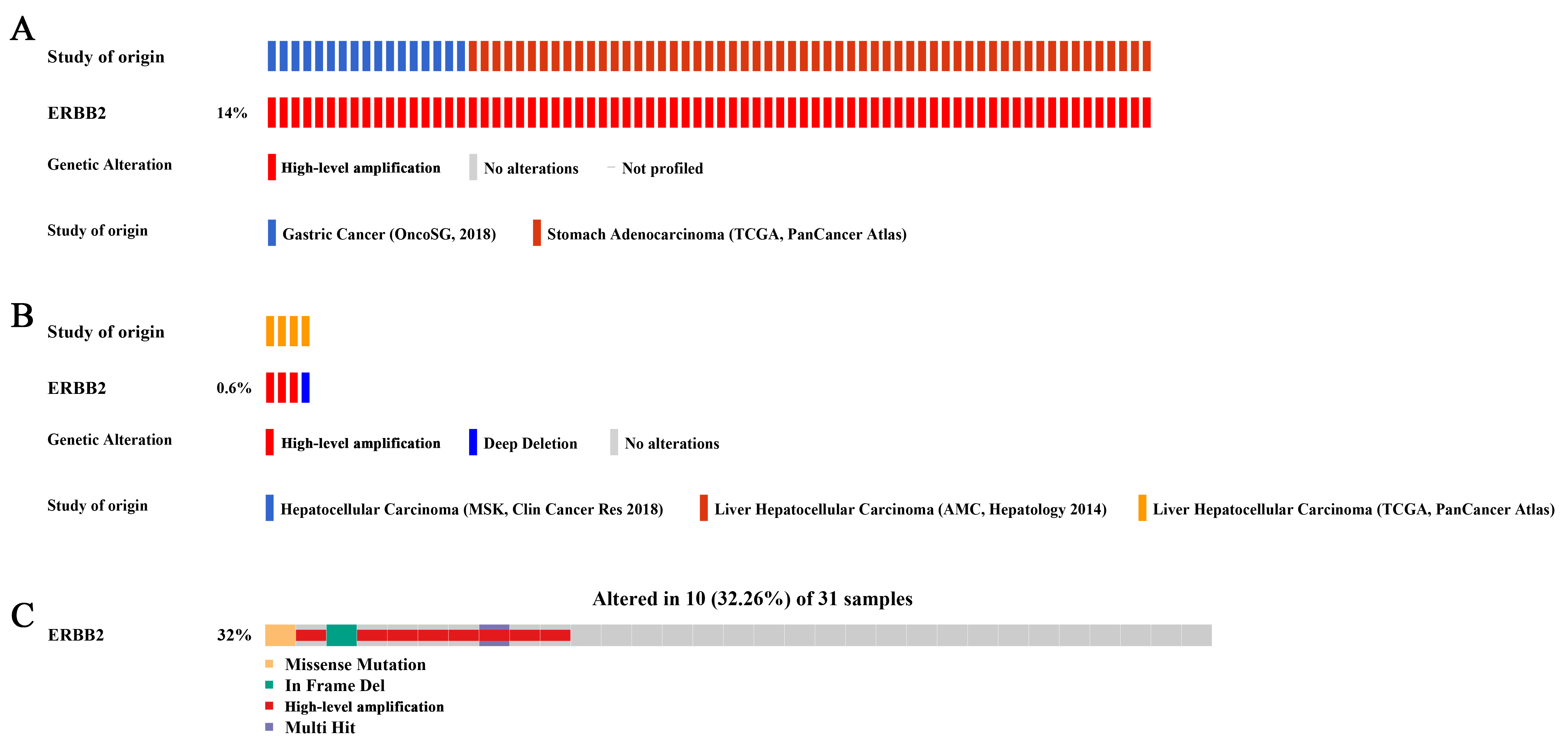

Supplement: Supplementary file 1 [file cancers-14-03849-s001.zip › Fig S10.tif]

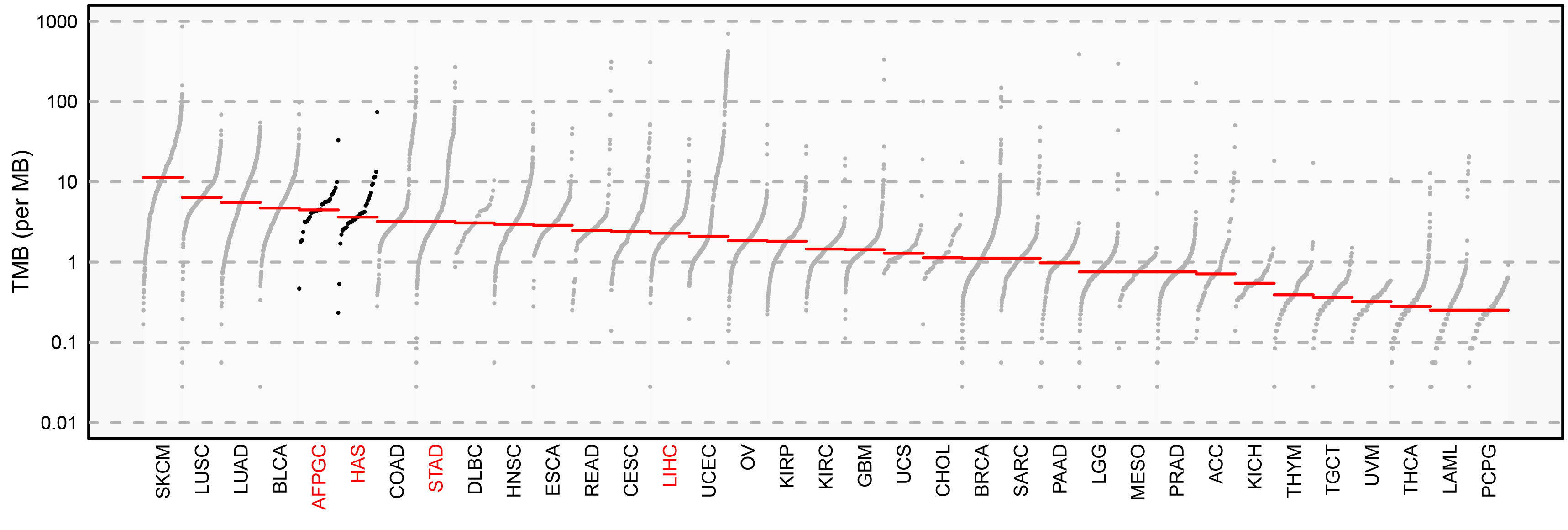

Supplement: Supplementary file 1 [file cancers-14-03849-s001.zip › Fig S11.tif]

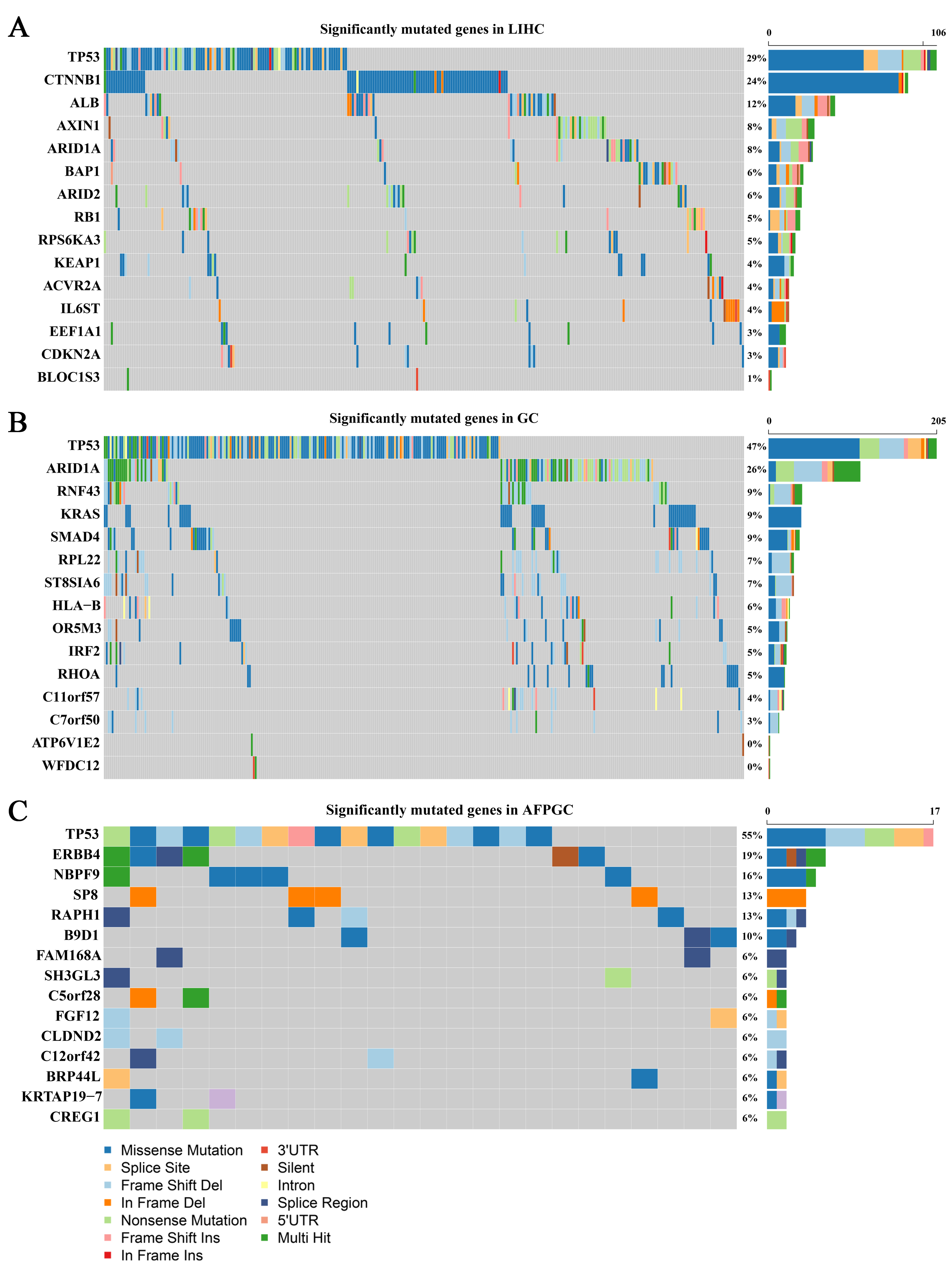

Supplement: Supplementary file 1 [file cancers-14-03849-s001.zip › Fig S2.tif]

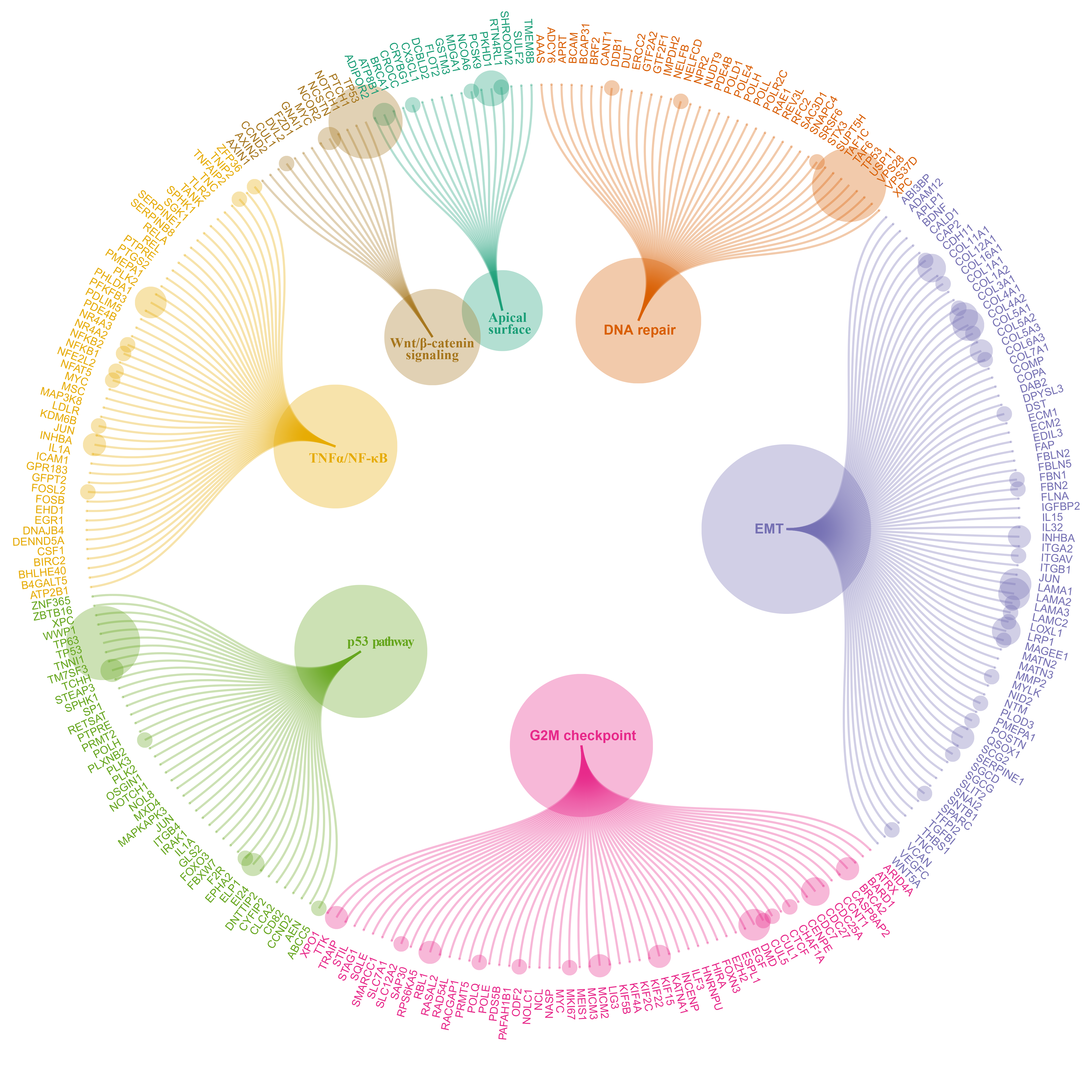

Supplement: Supplementary file 1 [file cancers-14-03849-s001.zip › Fig S3.tif]

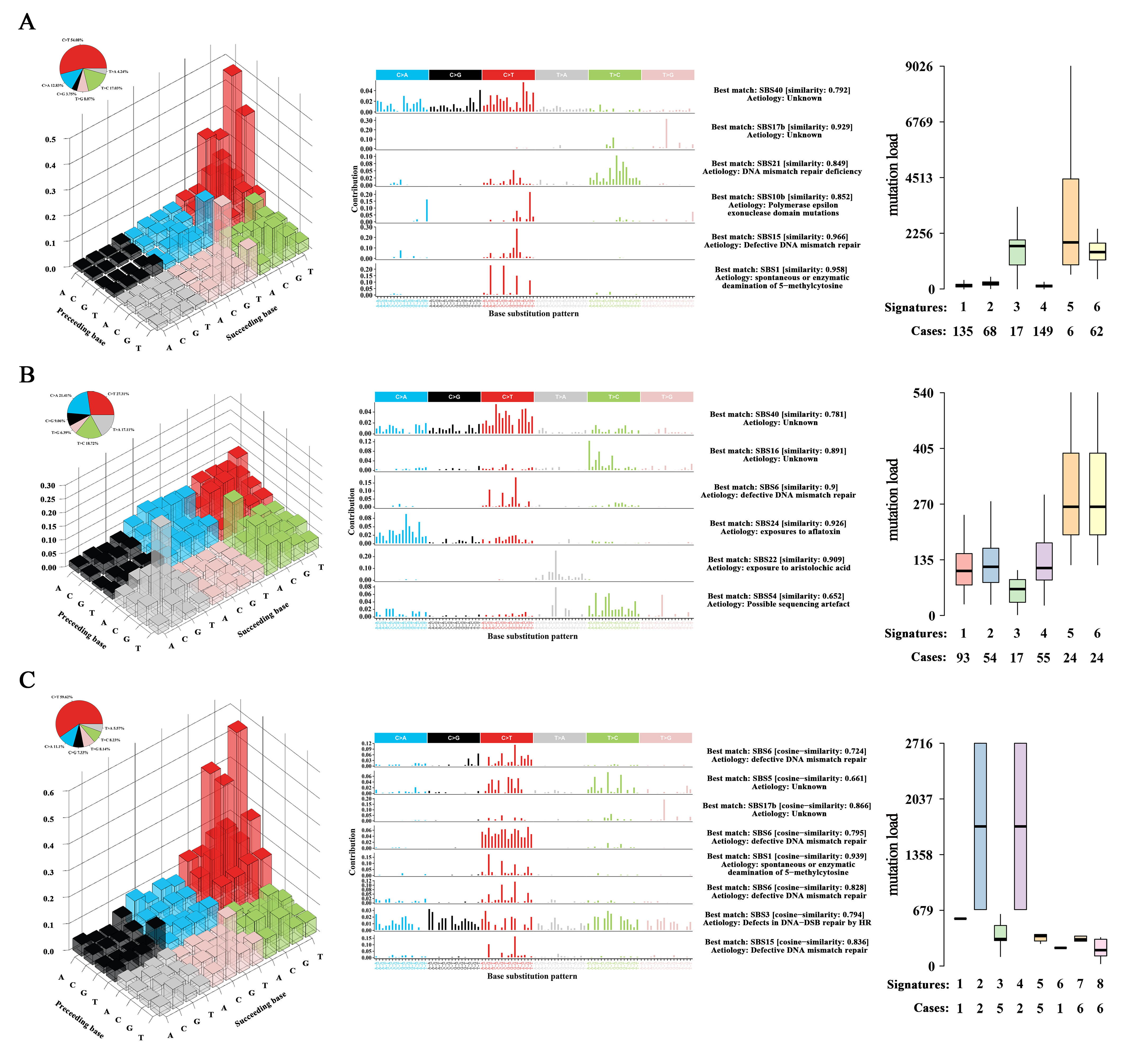

Supplement: Supplementary file 1 [file cancers-14-03849-s001.zip › Fig S4.tif]

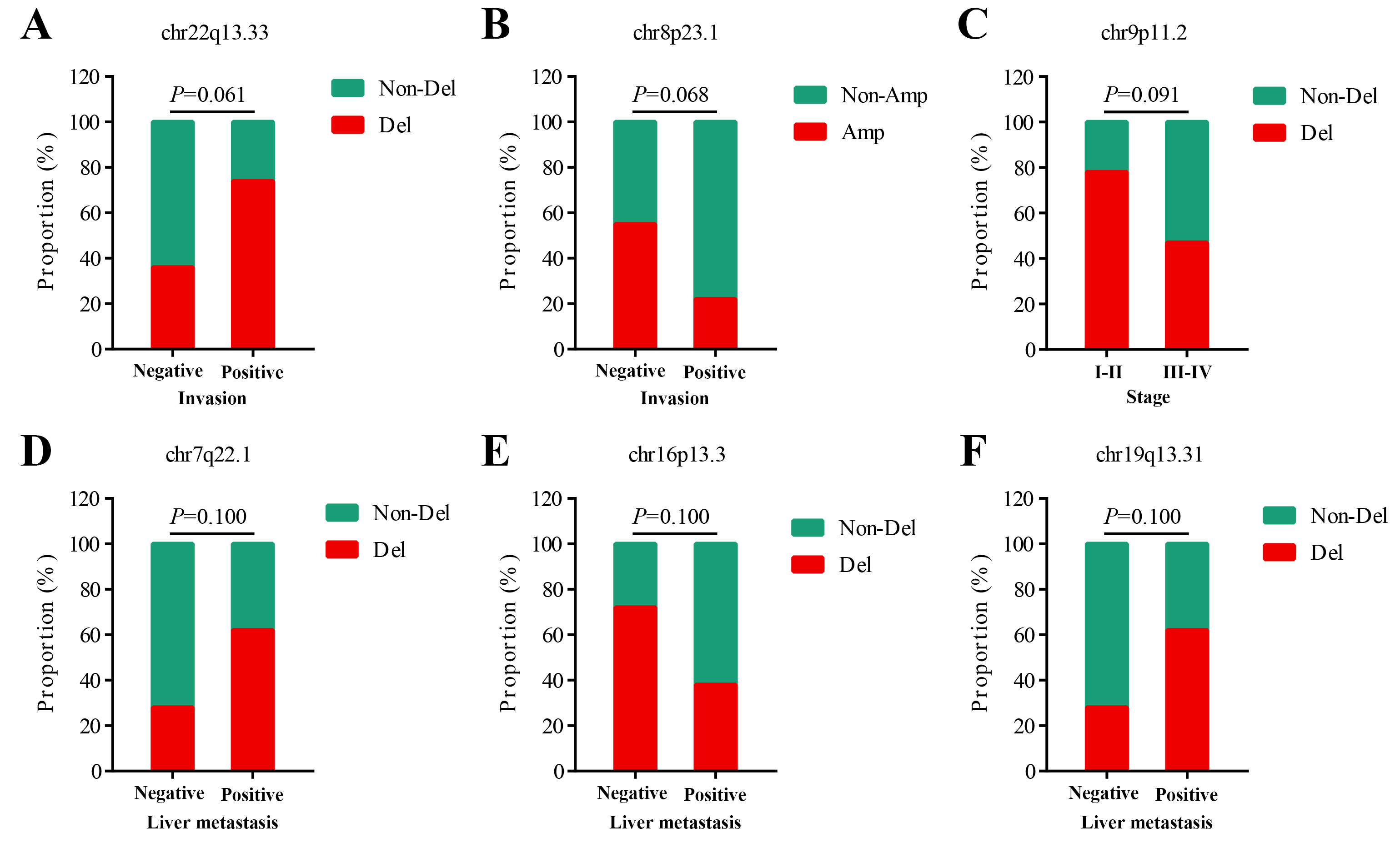

Supplement: Supplementary file 1 [file cancers-14-03849-s001.zip › Fig S5.tif]

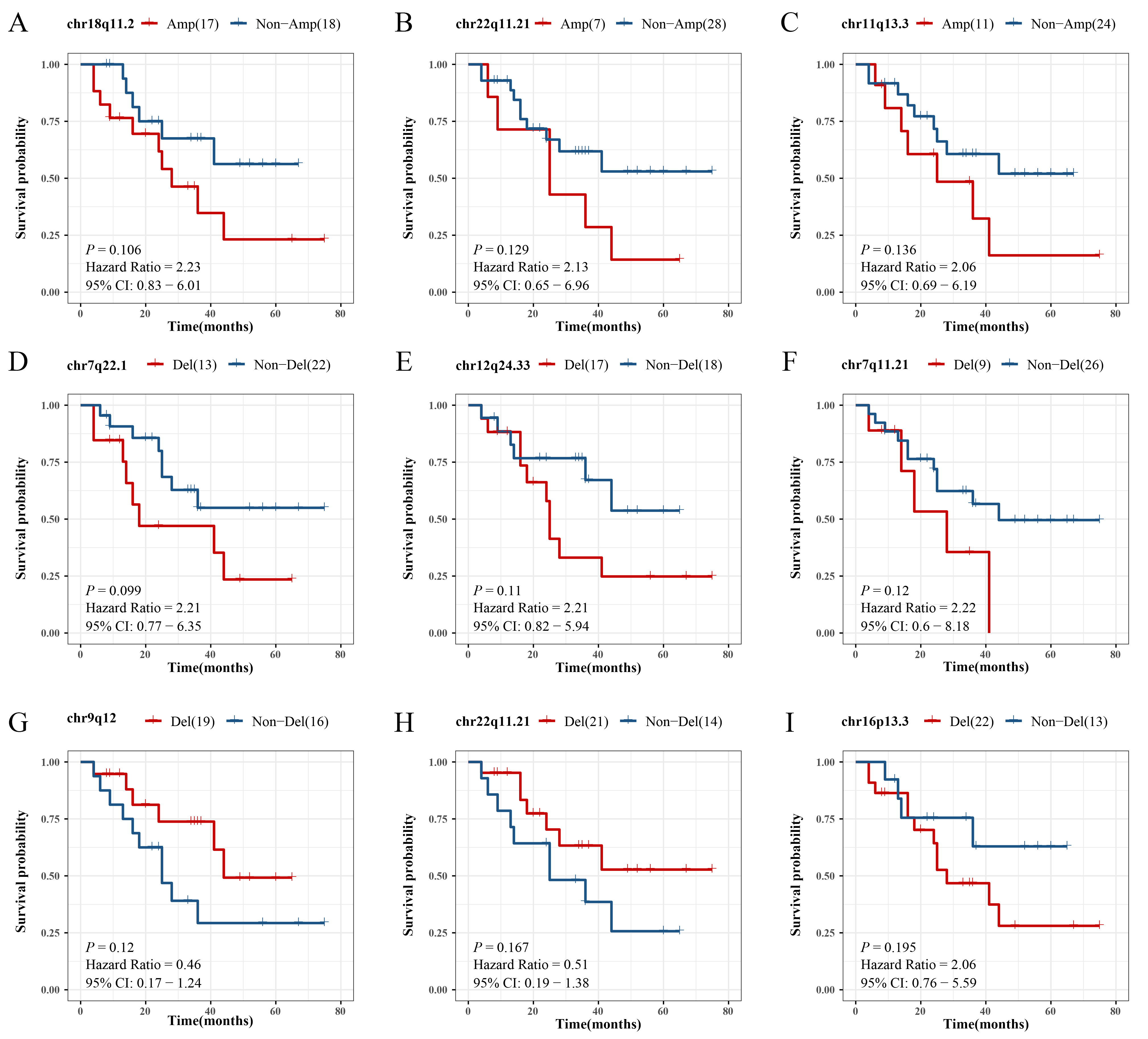

Supplement: Supplementary file 1 [file cancers-14-03849-s001.zip › Fig S6.tif]

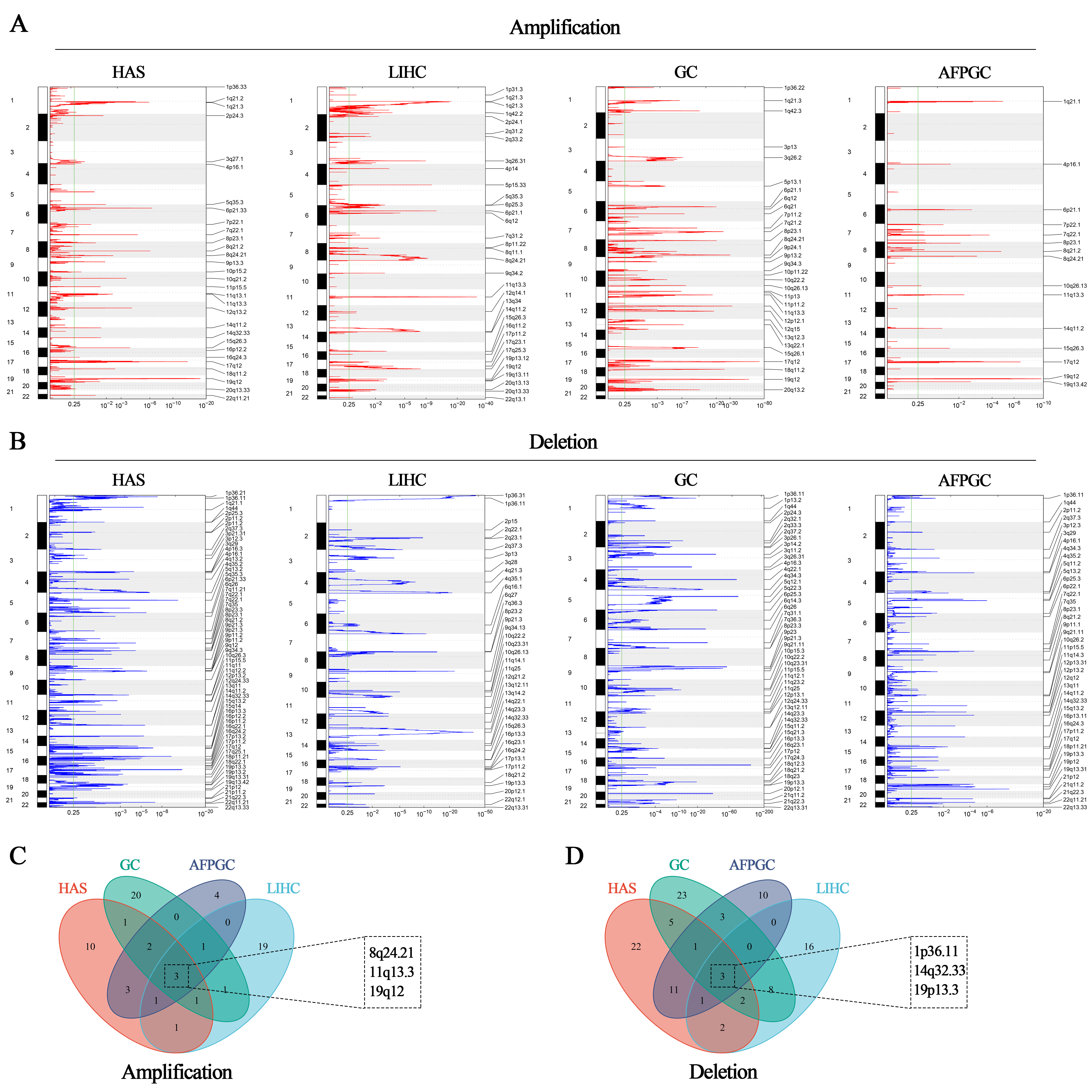

Supplement: Supplementary file 1 [file cancers-14-03849-s001.zip › Fig S7.tif]

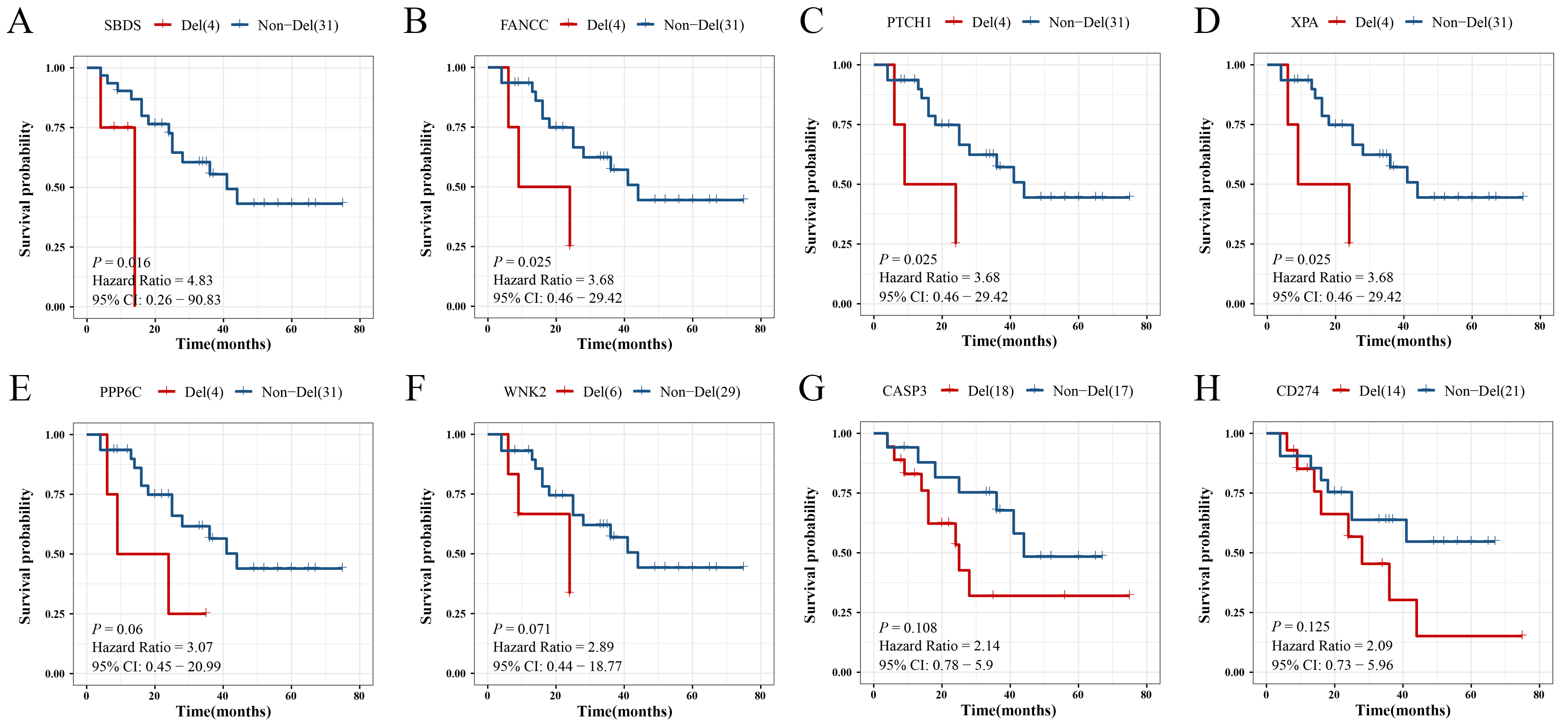

Supplement: Supplementary file 1 [file cancers-14-03849-s001.zip › Fig S8.tif]

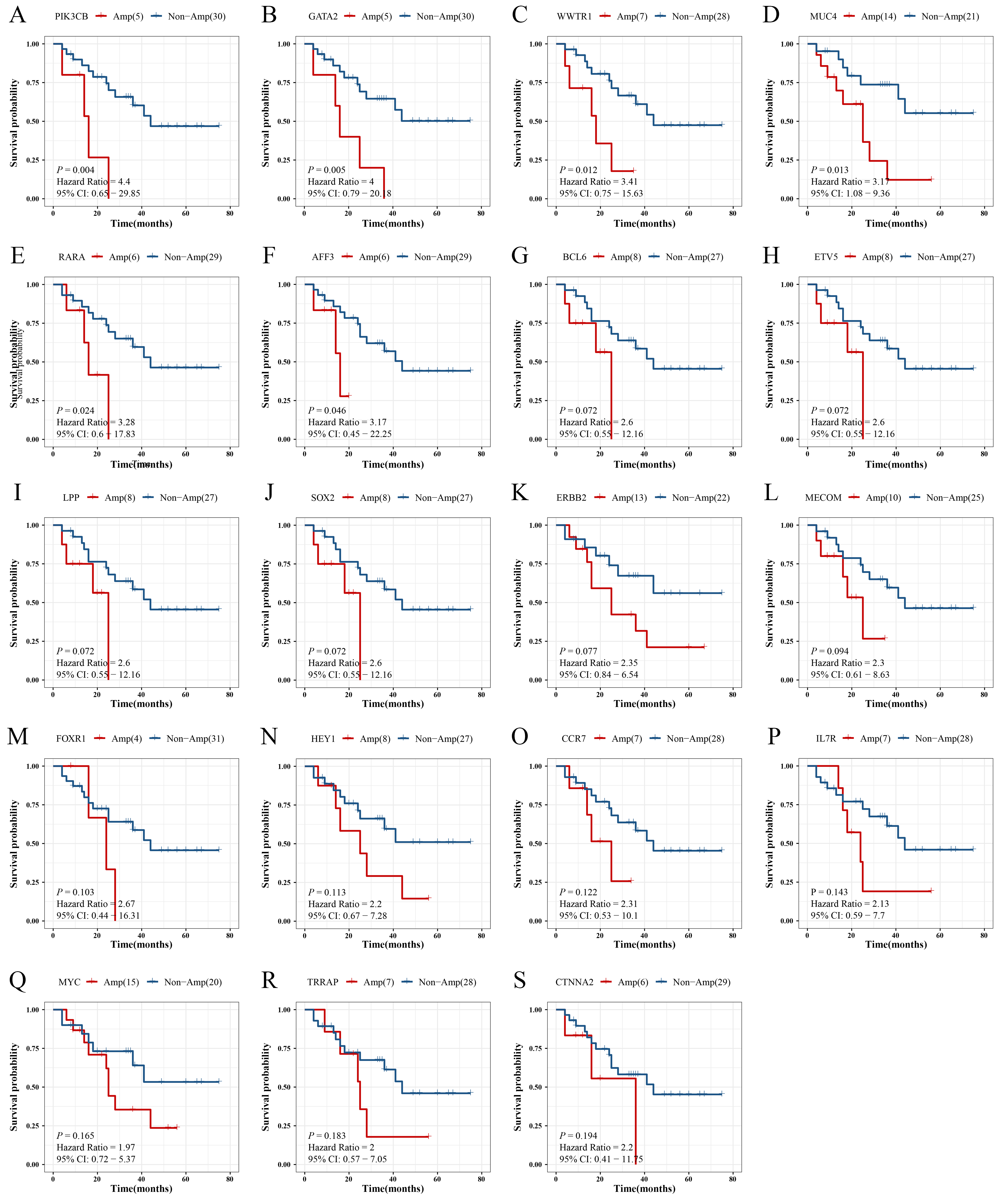

Supplement: Supplementary file 1 [file cancers-14-03849-s001.zip › Fig S9.tif]
